# Reconstruction and Validation of Arterial Geometries for Computational Fluid Dynamics Using Multiple Temporal Frames of 4D Flow-MRI Magnitude Images

**DOI:** 10.1007/s13239-023-00679-x

**Published:** 2023-08-31

**Authors:** Scott MacDonald Black, Craig Maclean, Pauline Hall Barrientos, Konstantinos Ritos, Asimina Kazakidi

**Affiliations:** 1 Department of Biomedical Engineering, University of Strathclyde, Glasgow, UK; 2 Research and Development, Terumo Aortic, Glasgow, UK; 3 Clinical Physics, Queen Elizabeth University Hospital, NHS Greater Glasgow & Clyde, Glasgow, UK; 4 Department of Mechanical and Aerospace Engineering, Glasgow, UK; 5 Department of Mechanical Engineering, University of Thessaly, Volos, Greece

**Keywords:** 4D Flow-MRI, CT, Aorta, Segmentation, Reconstruction, CFD

## Abstract

**Purpose:**

Segmentation and reconstruction of arterial blood vessels is a fundamental step in the translation of computational fluid dynamics (CFD) to the clinical practice. Four-dimensional flow magnetic resonance imaging (4D Flow-MRI) can provide detailed information of blood flow but processing this information to elucidate the underlying anatomical structures is challenging. In this study, we present a novel approach to create high-contrast anatomical images from retrospective 4D Flow-MRI data.

**Methods:**

For healthy and clinical cases, the 3D instantaneous velocities at multiple cardiac time steps were superimposed directly onto the 4D Flow-MRI magnitude images and combined into a single composite frame. This new Composite Phase-Contrast Magnetic Resonance Angiogram (CPC-MRA) resulted in enhanced and uniform contrast within the lumen. These images were subsequently segmented and reconstructed to generate 3D arterial models for CFD. Using the time-dependent, 3D incompressible Reynolds-averaged Navier–Stokes equations, the transient aortic haemodynamics was computed within a rigid wall model of patient geometries.

**Results:**

Validation of these models against the gold standard CT-based approach showed no statistically significant inter-modality difference regarding vessel radius or curvature (p > 0.05), and a similar Dice Similarity Coefficient and Hausdorff Distance. CFD-derived near-wall hemodynamics indicated a significant inter-modality difference (p > 0.05), though these absolute errors were small. When compared to the in vivo data, CFD-derived velocities were qualitatively similar.

**Conclusion:**

This proof-of-concept study demonstrated that functional 4D Flow-MRI information can be utilized to retrospectively generate anatomical information for CFD models in the absence of standard imaging datasets and intravenous contrast.

**Supplementary Information:**

The online version contains supplementary material available at 10.1007/s13239-023-00679-x.

## Introduction

Accurate representation of the arterial geometry and blood flow regime plays a fundamental role in clinical practice for disease diagnosis, staging, treatment planning, and patient outcome monitoring [1–3]. The reconstruction of the aorta and main branches, in particular, is inherently challenging due to a high variability in diameter, shape, and overall geometry within the healthy and patient population [4]. In patients wherein a stent-graft has been deployed, metal-induced artifacts within medical images can further complicate this process [5].

Computational tomography (CT) is the preferred imaging modality for arterial visualization and reconstruction in clinical practice, especially where stent-grafts are present [6–9]. However, CT scans utilize ionizing radiation, which is well-known to cause long-term health risks [10–12]. There is now a growing awareness in medical imaging to reduce radiation exposure where possible, especially in children and during asymptomatic screening [11, 13–21]. Additionally, ethical implications prohibit the use of CT to generate anatomical or functional reference models within the healthy population.

Magnetic resonance angiography (MRA), a subset of magnetic resonance imaging (MRI), is a non-ionizing alternative to CT imaging [6, 9, 22]. With the addition of intravenous contrast agents, the signal to noise ratio (SNR) and contrast to noise ratio (CNR) are increased significantly. However, in patients with high sensitivity to contrast agents, (e.g. with impaired renal function, which is common in those who require stent-grafts), the emergence of non-contrast MRA has been beneficial, including techniques such as time of flight (TOF), phase contrast (PC), and four-dimensional flow (4D Flow) [23–29].

4D Flow-MRI is a relatively recent development which captures the spatiotemporal evolution of 3D blood flow with full volumetric coverage throughout a continuous region of interest (ROI) at multiple cardiac time steps [30, 31]. In this time-resolved, respiratory and ECG-gated acquisition, velocity vectors are encoded in all three spatial dimensions, which permits post-hoc, dynamic visualization of the flow regime and quantification of several hemodynamic parameters [32]. Generally, arterial reconstruction from 4D Flow-MRI can be achieved through direct volume rendering within specific 4D Flow software, or via contour-based segmentation of 4D Flow-MRI derived MRA, comprised of 2D image stacks [33]. With volume rendering, the user has limited control over the final geometry, which may falsely include parts of the surrounding tissue or exclude regions of low velocities, e.g. in curvature and branching points. Contour-based segmentation grants increased user control and has previously been performed via supervised (convolutional neural networks) and unsupervised (k-means clustering) machine learning, atlas-based approaches, deformable model algorithms, and blood vessel tracking algorithms [4, 34–40]. To utilize these segmentation techniques, the data must first be processed into discretized image stacks.

Due to the retrospective nature of this study and data availability, the 4D Flow-MRI images were not accompanied by standard and well-established images such as MRA or PC-MRA. PC-MRA, for example, is commonly used to generate images for blood vessel reconstruction and does not require intravenous contrast [41]. These angiographic images can, however, be created directly from the retrospective 4D Flow-MRI data [41]. Due to the complexity of the 4D Flow-MRI data, translating this information into images which clearly portrays the underlying anatomical structures is challenging [42]. However, the ability to generate contrast within the lumen in these retrospective datasets and prepare images for segmentation is essential for subsequent reconstruction.

Therefore, the aim of this paper is twofold. The primary aim was to outline a novel methodology to create a phase contrast angiogram by retrospectively superimposing the instantaneous 4D Flow-MRI-derived velocity profile at multiple, user-defined time steps directly onto the magnitude image stack. As this methodology creates a composite image, the resultant dataset shall be termed as a Composite Phase-Contrast Magnetic Resonance Angiogram (CPC-MRA). To validate this approach, a study was undertaken to validate the 4D Flow-MRI reconstructed geometries against CT-based reconstructions from a healthy volunteer and clinical patients. The secondary aim was to evaluate haemodynamics from computational fluid dynamics (CFD) models, based on the reconstructed geometries from each imaging modality. This secondary study was to investigate whether minor changes in the reconstructed geometries had a large impact on CFD-derived haemodynamics. In each of the two studies, CT-imaging was utilized as the reference gold-standard approach. The CFD-derived velocities for each patient were then compared to that obtained directly from the 4D Flow-MRI data.

## Methodology

### Temporal Composite and Arterial Reconstruction

#### Imaging Datasets

4D Flow-MRI and CT data from three patients with arterial pathology in the thoracoabdominal region were acquired from the Queen Elizabeth University Hospital (QEUH). 4D Flow-MRI data was also obtained from a healthy volunteer (Table 1). Each clinical patient, hereafter termed patient 1, 2, and 3, was diagnosed with an abdominal Type B aortic dissection. Patient 1 had a previous Anaconda™ stent-graft deployed in the distal abdominal aorta, extending into the iliac bifurcation.

**Table 1:** Computed tomography (CT) and 4D Flow-magnetic resonance imaging (4D Flow-MRI) datasets obtained from a healthy volunteer and three clinical patients

	Age	Sex	CT	4D Flow-MRI	Clinical pathology
Volunteer	33	M	–	✓	–
Patient 1	68	M	✓	✓	Type B AD & Anaconda™ stent-graft
Patient 2	55	M	✓	✓	Type B AD
Patient 3	62	M	✓	✓	Type B AD

*AD:* aortic dissection

#### 4D Flow-MRI Scan Sequence

4D Flow-MRI images were acquired using an MRI research 4D flow sequence (WIP 785A), from Siemens: 80 × 160 × 60 mm^3^ imaging volume, 3.6 × 2.4 × 2.6 mm^3^ acquired resolution, TR/TE (Repetition Time/Echo Time) = 3.8/2.8 ms, integrated parallel acquisition technique (iPat) 3. Velocity encoding (VENC) was 150 cm/s, with a scan time of ~ 8 min and 20-time frames between each R-R interval. Contrast media was not utilized. The acquisition used retrospective electrocardiogram (ECG) gating and respiratory gating navigator. CT images were obtained via a contrast-enhanced CT angiography (CE-CTA) helical scan, with no cardiac gating, using iodinated contrast material (100 ml).

#### Blood Velocity Visualization

**Fig. 1:**
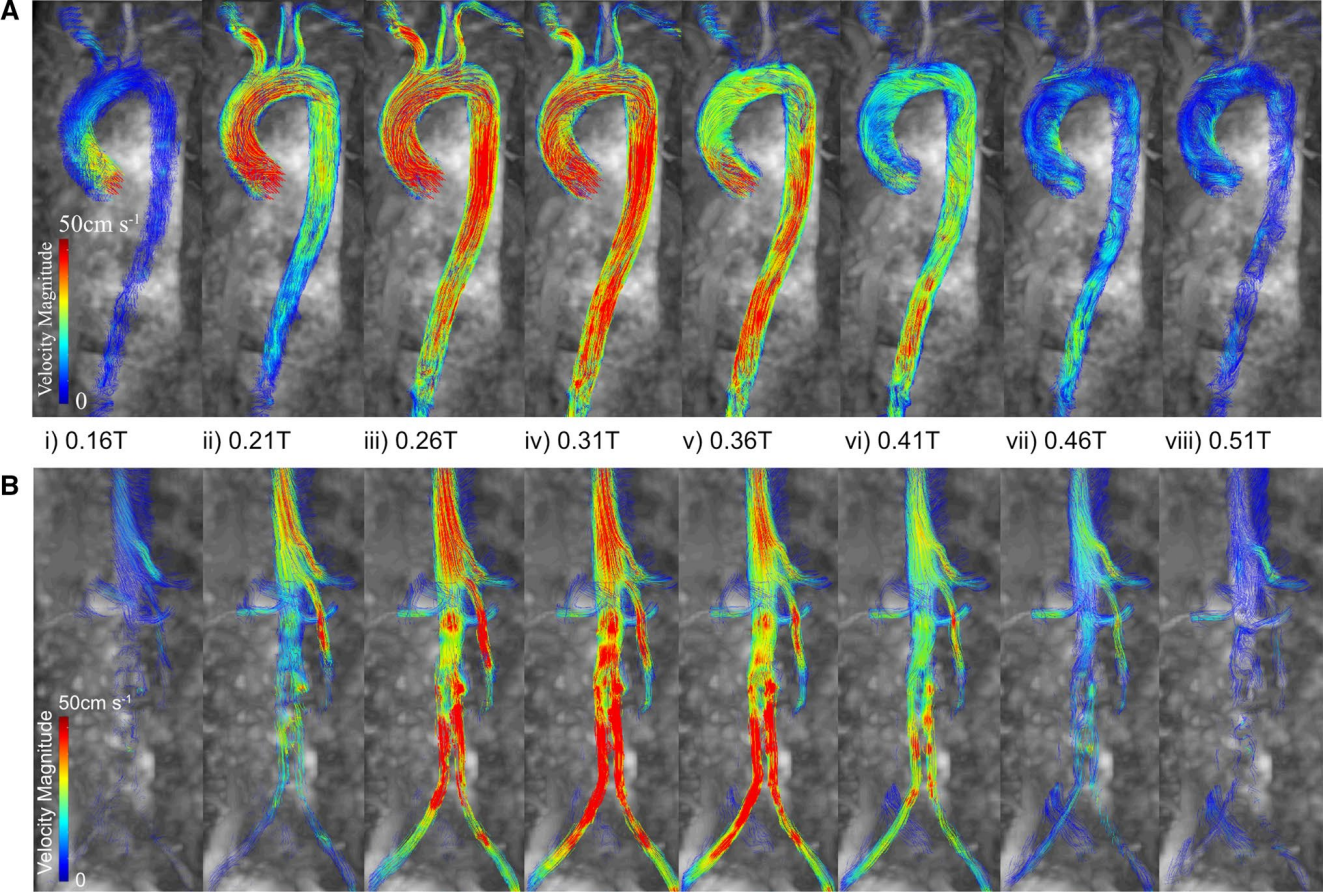
Velocity streamlines at consecutive time steps throughout **A** the thoracic aorta of a healthy volunteer, and **B** the abdominal aorta and common iliac arteries of patient 1 with an Anaconda™ stent-graft. This was obtained from analysis of 4D Flow-MRI data on circle cardiovascular imaging software, cvi42®. All images are shown between a velocity scale of 0–50 cm s^−1^ at time points throughout the cardiac cycle, where T is the cardiac period (0.21 T: Systolic acceleration (SA); 0.26 T: Peak systole (PS); 0.36 T: Systolic deceleration (SD))

The 4D Flow-MRI datasets were imported into Circle Cardiovascular Imaging Software (cvi42®, Calgary, Canada) and excess volume surrounding the aorta and its main branches was removed manually [43]. Thereafter, a mask (based on detected areas of flow) was applied to the 4D Flow-MRI magnitude images, and the threshold was set to ensure that the entirety of the aorta and branches were rendered. A mask correction was then employed to differentiate between static tissue, air filled regions, and regions of blood flow. From this mask, a rough, 3D volume render of the aorta was generated. To visualize the velocity streamlines, the aorta and main branches were isolated from the heart and surrounding vasculature using a vessel centerline, created from multiple user-defined control points within the lumen. Figure 1a illustrates the velocity streamlines in the thoracic aorta of the healthy volunteer throughout a single cardiac cycle, while Fig. 1b shows the abdominal aorta and iliac arteries of patient 1. It was evident that the instantaneous 3D velocity profile highlighted different regions of the lumen depending on the stage of the cardiac cycle. For example, the signal intensity in the ascending aorta, supra-aortic branches, and abdominal aorta was greatest during systolic acceleration (SA), peak systole (PS), and systolic deceleration (SD) respectively. Blood flow gradually became non-directional and low in magnitude as diastole was approached.

#### Geometry Reconstruction from 4D Flow-MRI

**Fig. 2:**
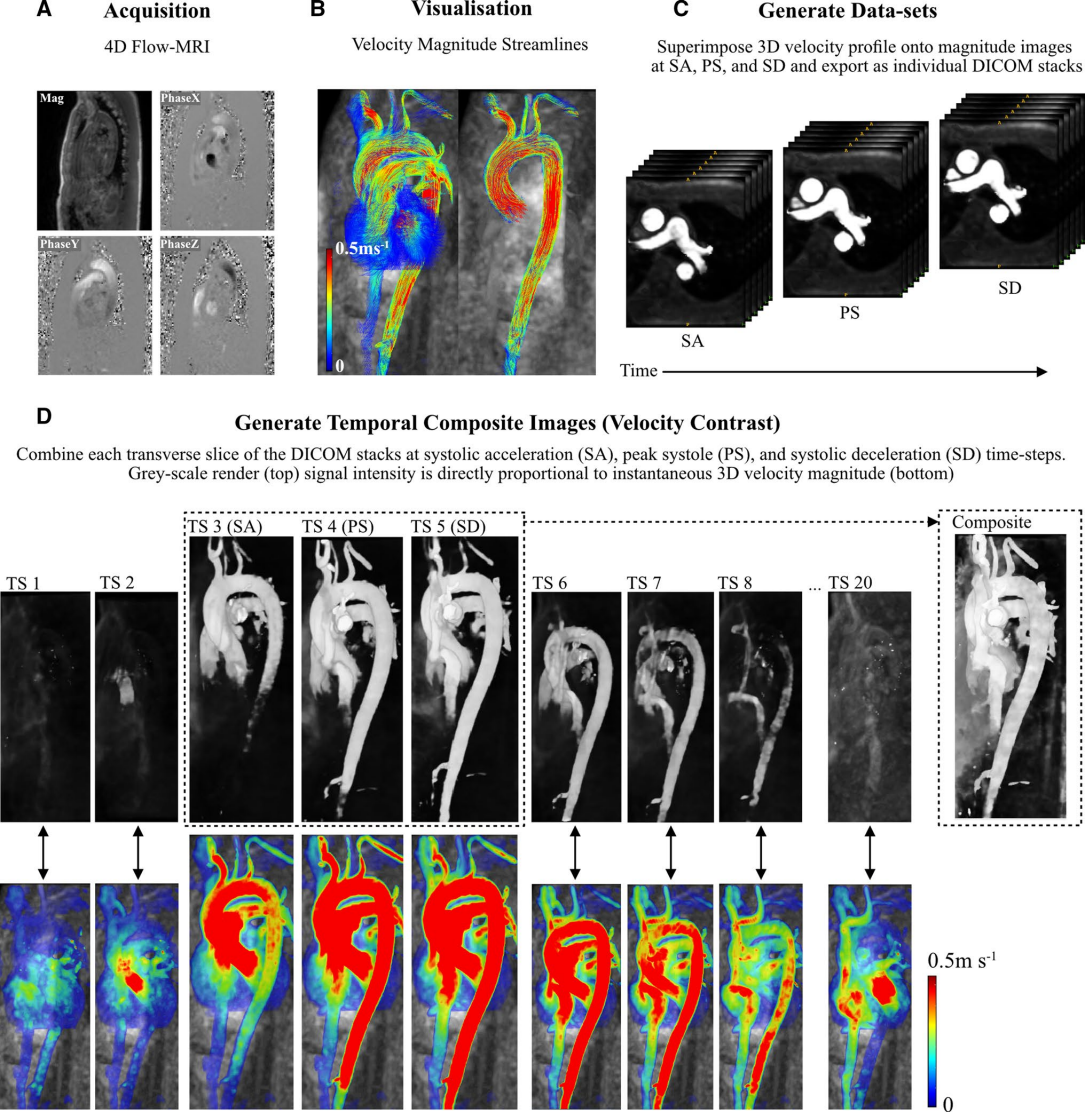
An illustration of the proposed CPC-MRA extraction from 4D Flow-MRI data of the thoracic aorta of the healthy volunteer. **A** 4D Flow-MRI data acquisition at the thoracic aorta. **B** 3D velocity encoding permitted analysis of velocity at any point in the region of interest (ROI), from which the aorta itself can be isolated for visualization. **C** The 3D velocity profile was superimposed directly onto the magnitude images and the ROI was discretized along the axial plane to create a DICOM stack at SA, PS, and SD. **D** The images at SA, PS, and SD were combined on a slice-by-slice basis to create CPC-MRA composite images in the axial, sagittal, and coronal plane. Velocity is directly proportional to signal intensity

Figure 2 outlines the process of geometry reconstruction from 4D-Flow MRI images from multiple user-defined time steps, at SA, PS, and SD. Centerlines were utilized only for streamline visualization (Fig. 2b) and removed thereafter. Subsequent steps in the methodology therefore encompassed blood flow through all vasculature within the ROI to reduce inter- and intra-user variability. For the healthy volunteer and clinical patient, the 3D instantaneous velocity profile of blood at multiple (SA, PS, SD), discrete time points were utilized to generate contrast within the vessel lumen. This data was extracted retrospectively from the 4D Flow-MRI data. To do this, the continuous ROI was first discretized to create a finite number of image slices in the transverse (axial) plane (n = 1200 slices), coronal plane (n = 500 slices), and sagittal plane (n = 500 slices) at SA, PS, and SD. At each of these three time steps, the discretized slices within the image stack were separated by a slice gap thickness of 0.35 mm (Fig. 2C), which was the minimum possible gap thickness which could be created on cvi42®. The final resolution was, however, limited to 3.6 × 2.4 × 2.6 mm^3^ due to the acquisition sequence. The 3D velocity profile at SA, PS, and SD, as calculated on cvi42®, was overlaid directly onto each image within the DICOM stack via superimposition. In regions of non-zero blood velocity (i.e. within the lumen), the velocity signal was converted to a greyscale image, where pixel intensity within each slice of the image stacks was proportional to the instantaneous velocity magnitude of blood (Fig. 2C). This generated additional contrast against the surrounding static tissue. The adopted velocity threshold for signal intensity for DICOM generation was 25 cm s^−1^. A composite DICOM image, hereafter termed a Composite Phase-Contrast Magnetic Resonance Angiogram (CPC-MRA) stack for each person was then created from images by combining the velocity-enhanced DICOM stacks (Fig. 2D) at SA, PS, and SD (i.e. $$SliceN_{CPC - MRA} = SliceN_{SA} + SliceN_{PS} + SliceN_{SD} )$$, where N is the slice number in the transverse, coronal, or sagittal plane. Crucially, superimposition and alpha blending were used to combine the images from each time step to ensure uniform intensity within the lumen. The contrast was then enhanced on the final DICOM stack by re-mapping the intensity values in the initial grayscale image to new values to fill the entire available intensity range using the Matlab® *imadjust* function [44]. This process of combining the image stacks was performed with an in-house Matlab® script (https://doi.org/10.15129/2db504b8-3736-4ba0-9829-b7cc0c5db38a) and is shown in Fig. 2D for a single slice of the thoracic aorta.

**Fig. 3:**
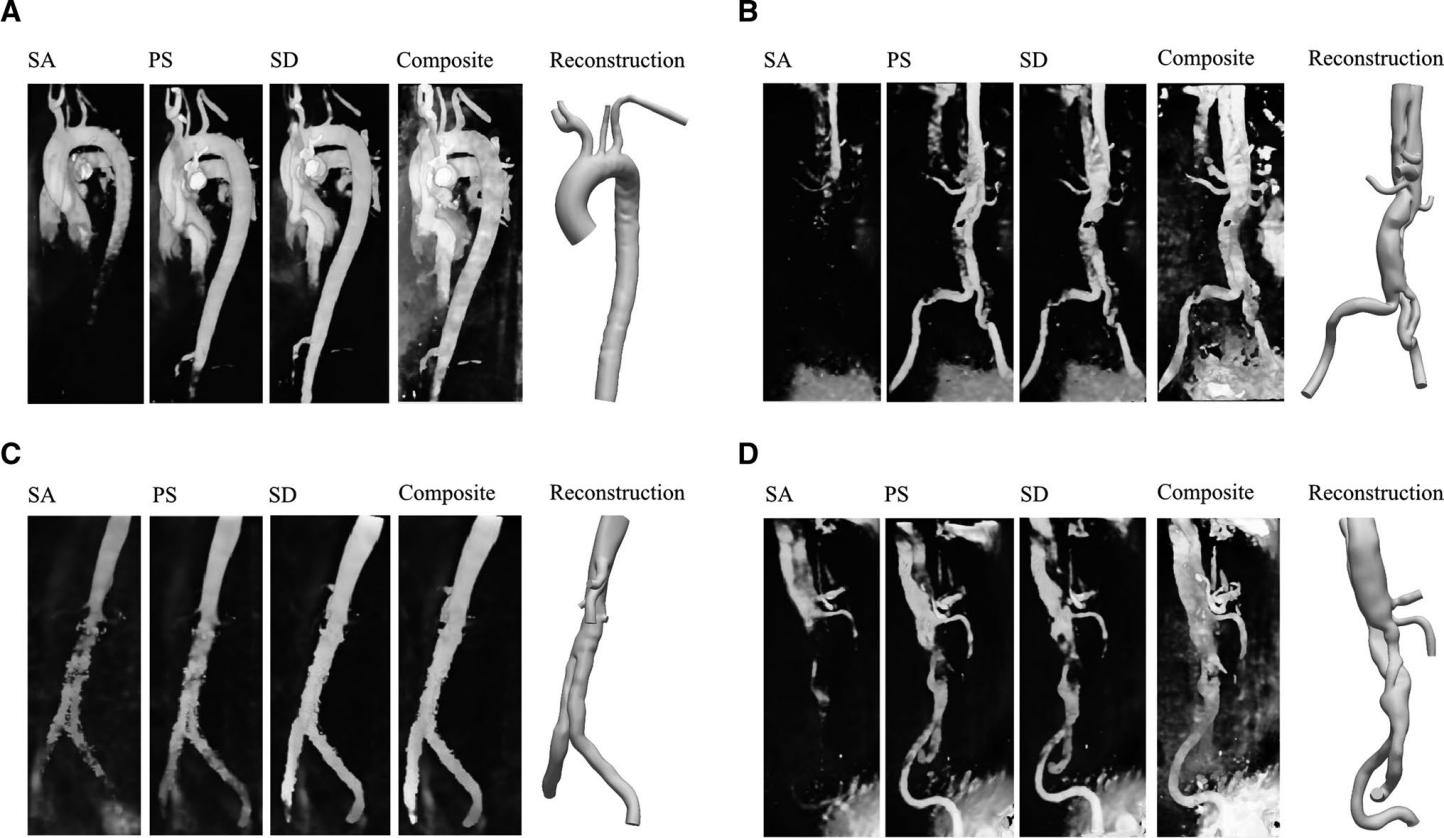
3D greyscale render of the velocity-derived contrast at SA, PS, and SD, the 4D Flow-MRI CPC-MRA, and the final SimVascular reconstruction for the **A** healthy volunteer, **B** patient 1, **C** patient 2, and **D** patient 3

The rationale behind the CPC-MRA images was as follows: if the user was to utilize only one time step, the resultant diameter in more distal regions of the aorta would be underestimated due to the temporal lag in arterial pulse waves which exhibit a reduced velocity and therefore reduced contrast. SA, PS, and SD were chosen as they generated the greatest degree of contrast throughout the entire aorta and branches when combined. Blood flow at previous and subsequent time steps was too low in magnitude to generate sufficient contrast for segmentation of the lumen. For each clinical patient and the healthy volunteer, the CPC-MRA DICOM stacks (generated from SA, PS, and SD) were then imported into the open-source software SimVascular® (https://simvascular.github.io/) [45]. Path lines were generated for each branch vessel via user-defined control points, whereafter Fourier smoothing was performed. 2D segmentations were created via manual image intensity thresholding to determine the vessel lumen contours along each path. To create a solid 3D model (Fig. 3), a lofted surface was generated based on the group of 2D segmentations along each vessel path. Finally, the lofted surfaces for each branch vessel were stitched together to create a single solid model which was subsequently smoothed (10 iterations of constrained smoothing and decimation). The reconstructed geometries were compared with the 4D-Flow MRI and CT images to ensure that the surface smoothing had no or minimal effect on the lumen dimensions.

### Validation with Computed Tomography

**Fig. 4:**
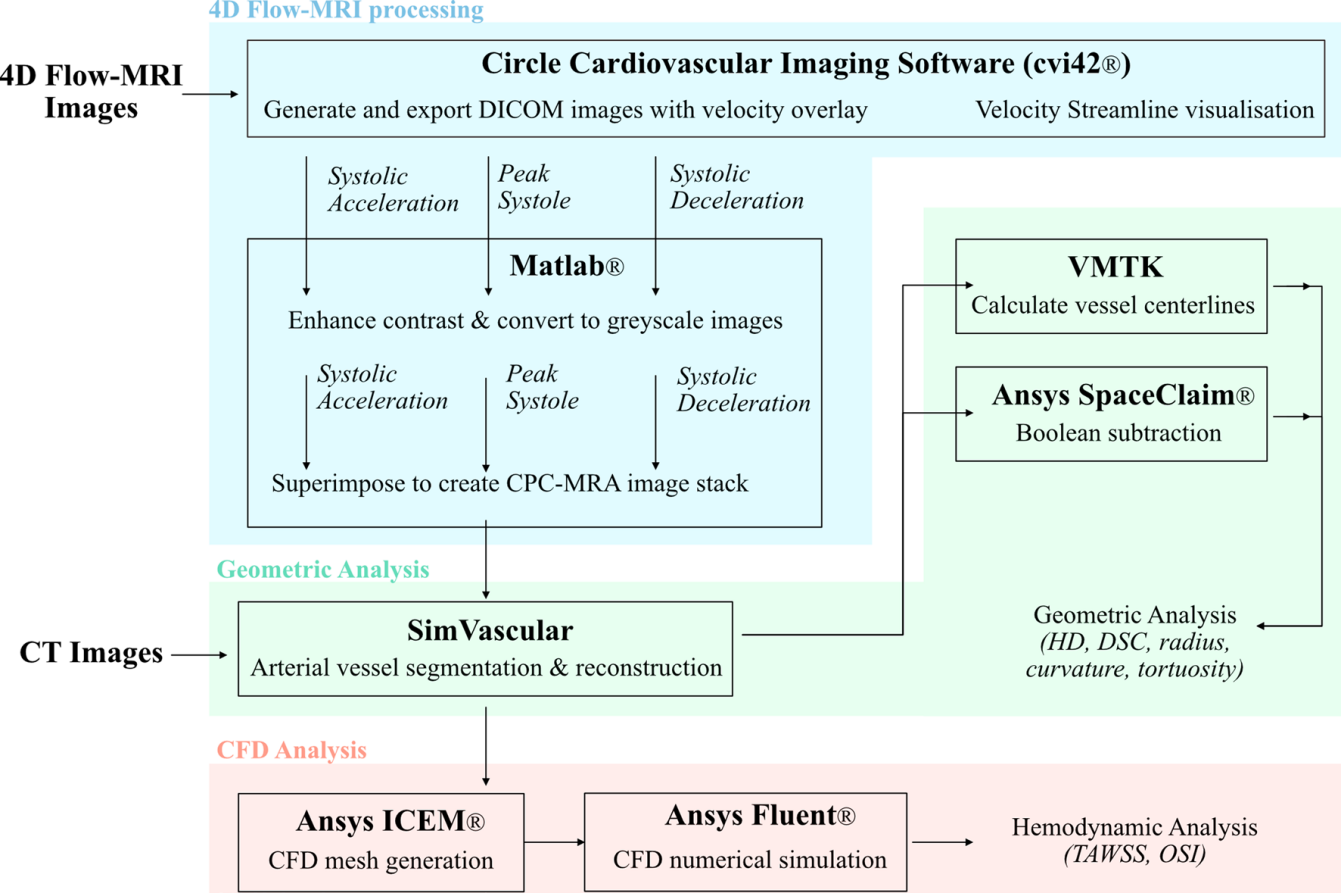
Flow chart to highlight the processing of the 4D Flow-MRI images to create the temporal composite (CPC-MRA) image stacks (blue) and subsequent geometric analysis of the 4D Flow-MRI and CT-derived reconstructed models (green), and CFD analysis (orange)

The 4D Flow-MRI based reconstruction methodology required validation against CT-derived models. As CT images were only available for clinical patients, this methodology was validated using the three clinical cases outlined in Table 1. CT and 4D Flow-MRI scans were performed on the same date for each patient. This validation comprised of five elements: (1) qualitative visual comparison of the 3D reconstructed arteries; (2) quantitative comparison of vessel centerline metrics including (i) *Radius:* Maximum inscribed sphere radius, (ii) *Curvature*: Inverse of the radius of the osculating circle [46], (iii) *Tortuosity*: The relative increment in length of a curve deviating from a straight line [46], and (iv) bifurcation angle; (3) *Hausdorff distance (HD):* The difference between two geometries by measuring their mutual proximity and the maximal distance between corresponding points of one relative to the other [47]; (4) *Dice Similarity Coefficient (DSC):* A spatial overlap index reflecting both size and localization agreement [48] ; and (5) quantitative comparison of near-wall hemodynamics from CFD simulations. Figure 4 illustrates this process as a flow chart.

Quantification of the inter-modality differences between the CT and 4D-Flow MRI-derived models was performed at the iliac bifurcation and common iliac arteries. This region provided a reference point common to both modalities and remained generally free from dissection. These aspects were important as the specific 4D Flow-MRI sequence utilized was not optimized for the analysis of a false lumen or small vessels. Bifurcations also generate complex hemodynamics and are inherently challenging to reconstruct, so geometric and hemodynamic comparisons at this region permit a robust analysis to be performed.

Curvature, κ(s), of the centerline, c(s), was defined as shown in Eq. 1 [46].

1 $$\kappa \left( s \right) = \frac{{\left| {c^{\prime}\left( s \right) \times c^{\prime\prime}\left( s \right)} \right|}}{{\left| {c^{\prime}\left( s \right)} \right|^{3} }}$$

As the arc length, L, of the centerline and the Euclidean distance between the end points, D, was known, tortuosity, $\chi$, was calculated as per Eq. 2 [46].

2 $$\chi = \frac{L}{D} - 1$$

If A represents the 4D Flow-MRI derived geometry and B represents the CT-derived geometry, the mathematical formulation for HD, H(A,B), is given in Eq. 3 [49]. As HD is a measure of boundary similarity between two objects, a comparison between two identical objects would result in a HD of zero.

3 $$H\left( {A,B} \right) = \max \left( {h\left( {A,B} \right),h\left( {B,A} \right)} \right)$$

where

4 $$h\left( {A,B} \right) = \mathop {\max }\limits_{a \in A} \left( {\mathop {\min }\limits_{b \in B} d\left( {a - b} \right) } \right)$$

5 $$h\left( {B,A} \right) = \mathop {\max }\limits_{b \in B} \left( {\mathop {\min }\limits_{a \in A} d\left( {b - a} \right) } \right)$$

where d represents the Euclidean distance between the points of the sets, h(A,B) is the forward HD, and h(B,A) is the backwards HD [49]. To ensure the 4D Flow-MRI and CT-derived models were oriented in the same plane, two points in the centerlines, the bifurcation reference point and the end point of the left iliac artery, were aligned**.** The HD between the boundaries of the 4D-Flow MRI and CT derived models was then analyzed at equally spaced, horizontal 2D planes (n = 1000) throughout the reconstructed models. The gap between each discrete plane was extremely small, so this tended towards a continuous analysis over the full model. The 95th percentile of the HD was utilized to handle outliers [50].

The formulation for the DSC is given in Eq. 6 [48]. DSC ranges from 0, indicating no spatial overlap between the 3D models, and 1, indicating a complete overlap [48].

6 $$DSC\left( {A,B} \right) = \frac{{2\left| {A \cap B} \right|}}{\left| A \right| + \left| B \right|}$$

When applied to discrete data, $\left| A \right|$ and $\left| B \right|$ are the cardinalities (number of elements) of the two sets, and ∩ is the intersection. Therefore, to compute the DSC, the cardinalities of the 4D Flow-MRI and CT-derived models, and the respective Boolean intersection $\left| {A \cap {\text{B}}} \right|$, for each patient were generated in Ansys SpaceClaim®.

The open-source Vascular Modelling Toolkit (VMTK) was used to compute vessel centerlines [51]. These centerlines were then resampled at 3 mm intervals and smoothed with a factor of 0.5 and 100 iterations to remove noise which can generate localized parameter errors. A bifurcation reference system was generated within the software, following the methodology of Piccinelli et al. [46]. Thereafter, bifurcation angles were obtained for each geometry, calculated from the difference between the in-plane angle of the common iliac vessels [52].

The abdominal aorta was truncated immediately upstream of the bifurcation reference point for both the 4D Flow-MRI and CT-derived models. This was to ensure that all 3D models began at a common anatomical landmark. Centerline measurements were performed distally to the bifurcation reference point. Patient 2 exhibited a small region of dissection in the left common iliac artery, resulting in a true lumen (TL) and false lumen (FL), so radius, R, was presented as R = R_TrueLumen_ + R_FalseLumen_.

#### Computational Fluid Dynamics Model

The 4D Flow-MRI and CT-derived models for patients 1, 2 and 3 were discretized to create a tetrahedral computational mesh in Ansys ICEM®. To capture the viscous sublayer, the initial prism layer height ($\Delta y_{1}$) on the boundary was estimated at $\Delta y_{1}$=2.5e−3 m, such that y +  < 1 [53]. To resolve the boundary layer, 10 prism layers were generated from this initial estimate, with an expansion ratio of 1.25. Further information on y + can be found in the Supplementary Material.

Grid convergence was then established for wall shear stress by performing several steady state Reynolds-averaged Navier Stokes (RANS) simulations in Ansys Fluent®, employing a shear stress transport (SST) k-ω turbulence model (Supplementary Material) [54]. The shear stress distribution was analyzed at the iliac bifurcation upon convergence of the solution, and a surface integral over the entire geometry was performed to yield a single quantitative metric. Upon satisfying grid convergence, the RANS output simulation results were examined to ensure the mesh was compliant with the y + criteria. Flow extensions were applied at the inlet (5D) and outlet (10D), where D was the inlet diameter of the respective patient vessel [55].

Transient aortic haemodynamics were computed within a rigid wall model with a no-slip boundary condition. Blood flow was modelled by solving the time-dependent, 3D, incompressible RANS equations for continuity and momentum, according to the following equations, respectively [56]:

7 $$\frac{{\partial \overline{u}_{i} }}{{dx_{i} }} = 0$$

8 $$\frac{{\partial \overline{u}_{i} }}{\partial t} + \overline{u}_{j} \frac{{\partial \overline{u}_{i} }}{{\partial x_{j} }} = - \frac{1}{\rho }\frac{{\partial \overline{p}}}{{\partial x_{i} }} + v\frac{{\partial^{2} \overline{u}_{i} }}{{\partial x_{j} \partial x_{j} }} - \frac{{d\tau_{ij} }}{{dx_{j} }}$$

where u is the fluid velocity, p is the pressure, ρ is the fluid density, and *v* is the kinematic viscosity. Reynolds decomposition was performed to separate velocity and pressure, into mean ($\overline{u}$, $\overline{p}$*)* and fluctuating ($u^{\prime}$, $p^{\prime}$) components, such that u = $\overline{u} + u^{\prime}$, p = $\overline{p} + p^{\prime}$, and $\tau_{ij} = \overline{{u_{i}^{\prime} u_{j}^{\prime} }}$ [56].

**Fig. 5:**
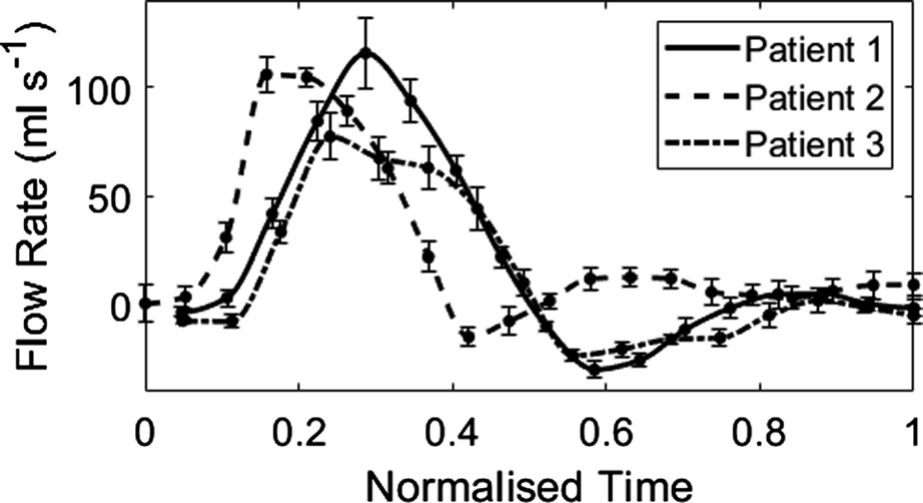
4D Flow-MRI derived flow waveforms extracted from the abdominal aorta, immediately proximal to the iliac bifurcation. At each time point (n = 20) throughout the cardiac cycle, the cross-sectional flow rate was calculated from 5 planes of analysis for each patient. These planes were equally spaced in the axial direction to discretely sample a volume of blood flow, from which a mean flow rate could be calculated. For each patient, this flow rate is presented as mean ± sd. Flow rate was then interpolated between each time point with a cubic function to generate a waveform with a time step of 0.001 s for CFD analysis

The governing equations were solved numerically via a finite volume method on Ansys Fluent® through a Pressure-Implicit with Splitting of Operators (PISO) algorithm with a second-order upwind scheme [57]. On average, each simulation required around 13 hours for 5 cardiac cycles on 35 Intel(R) Xeon(R) Gold 6138 CPU cores. Due to high shear rates within the aorta, blood was assumed to be a Newtonian fluid, with density 1060 kg m^−3^ and dynamic viscosity, μ, 0.004 Pa s [58, 59]. Time-periodicity was reached after five consecutive cardiac cycles for the CT and 4D Flow-MRI-derived models. For each patient, a 4D Flow-MRI derived flow waveform (Fig. 5) was extracted immediately upstream of the bifurcation reference point, i.e., the same location at which the 3D geometries were truncated. This flow waveform was then converted to a parabolic velocity profile (dt = 0.001 s) [60]. To ensure the shape, phase and peak of the inlet profile was consistent for numerical analysis, these velocity profiles were prescribed at the inlet of the computational domain for both the 4D Flow-MRI and CT-derived CFD models. Raw inlet flow rate data is presented as mean ± sd over 5 planes of analysis for each patient (Fig. 4). The mean and peak (mean/peak) Reynolds number (Re = ρUD/μ [61]) in the iliac arteries for patients 1, 2 and 3 was 453/1397, 389/1246, and 436/1260, respectively. At each outlet, a flow weighting was prescribed for the left and right (left/right) iliac arteries as 0.47/0.53, 0.65/0.35, and 0.53/0.47, respectively for patients 1, 2, and 3.

Haemodynamic analysis was performed only on the 5th cycle upon achieving a time-periodic solution, where the time-averaged wall shear stress (TAWSS) and OSI were investigated [62], defined as:

9 $$TAWSS = \frac{1}{T}\mathop \int \limits_{0}^{T} \left| {\vec{\tau }_{\omega } } \right|dt$$

10 $$OSI = \frac{1}{2}\left( {1 - \frac{{\left| {\mathop \int \nolimits_{0}^{T} \vec{\tau }_{\omega } dt} \right|}}{{\mathop \int \nolimits_{0}^{T} \left| {\vec{\tau }_{\omega } } \right|dt}}} \right)$$

where $\vec{\tau }_{\omega }$ represents the instantaneous wall shear stress vector, and T is the time for one cardiac cycle [62]. The upper 5% and lower 5% of TAWSS and OSI (N_Elements_ ~ 1100) were compared between modalities for each patient. These extremes were chosen for analysis since elevated TAWSS can be indicative of platelet activation and thrombus formation, while low and oscillatory regions can create stagnant flow and graft limb occlusion [63].

Finally, based on the normalized vessel centerlines and CFD simulations, a correlation and Bland-Altman plot for CT and 4D Flow-MRI derived vessel radius, curvature, TAWSS, and OSI was generated for each patient.

#### Sensitivity Analysis

An intra-user dependence study was performed to investigate the variability in arterial reconstruction resulting from manual segmentation of a single user. Thus, the CT and 4D Flow-MRI data for patient 3 were repeatedly segmented and reconstructed 5 times on SimVascular® by a single user. For each model, the previously described methods in Section 2.2 and 2.3 were employed to investigate vessel radius, curvature, and near-wall hemodynamics. To facilitate these simulations, only the forward flow was considered at the inlet to reduce computational demand.

## Results

### Reconstruction of Healthy Aortae and Great Vessels from 4D Flow-MRI

**Fig. 6:**
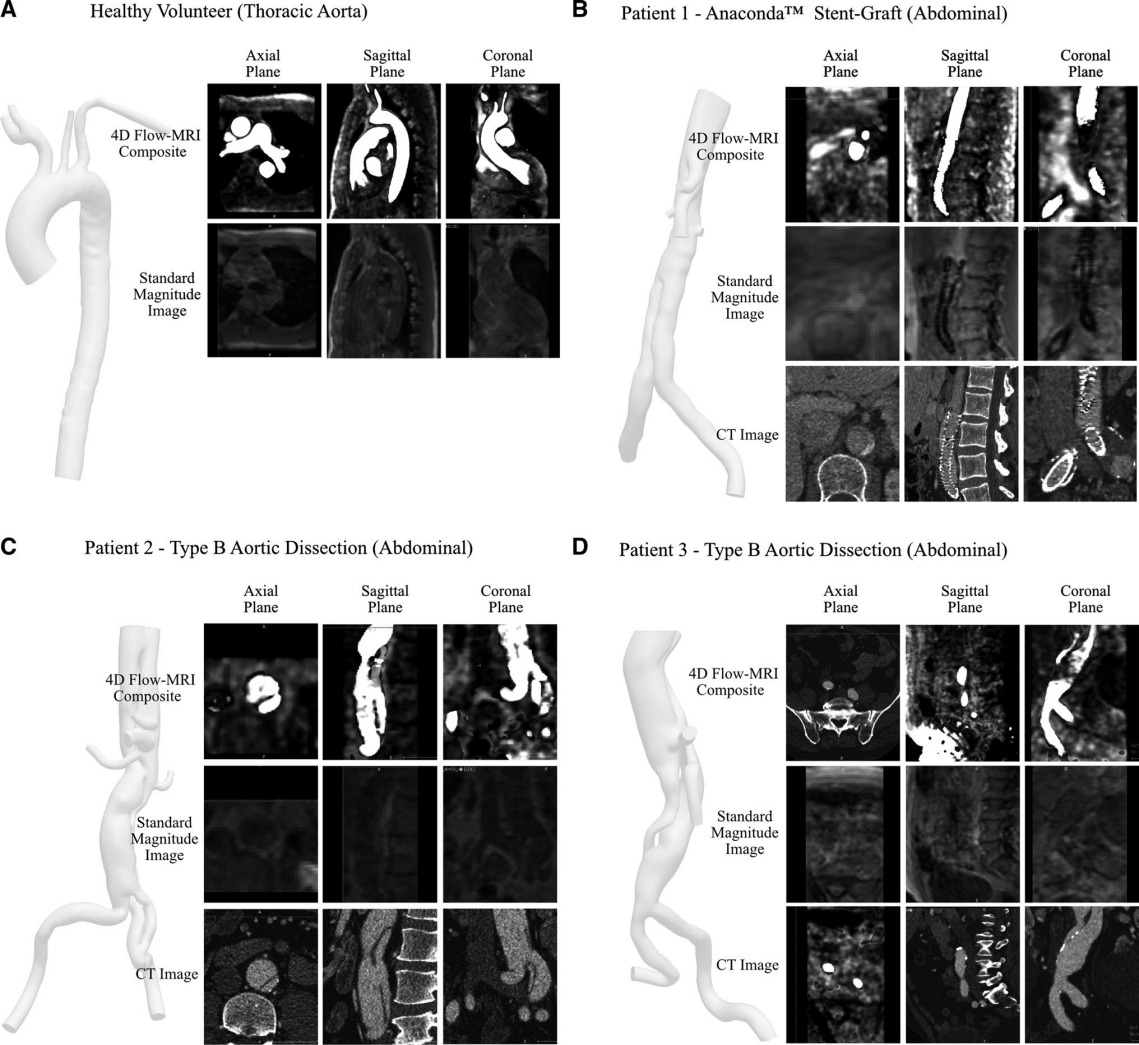
A computational model of **A** the thoracic aorta, reconstructed from the healthy volunteer, and the abdominal aorta and common iliac arteries, reconstructed from the 4D Flow-MRI CPC-MRA images for **B** patient 1, **C** patient 2, and **D** patient 3. The 3D models were created from the 4D Flow-MRI CPC-MRA images. Slices from the transverse, sagittal, and coronal planes from each case illustrate the arterial lumen from each DICOM dataset (CT, standard MRI magnitude image, 4D Flow-MRI CPC-MRA). CT images were not available for the healthy volunteer due to ethical considerations. The stent struts of patient 1 are not visible in the reconstructed model as 4D Flow-MRI yields data only on the flow lumen

Figure 6 illustrates the 4D Flow-MRI-derived model of volunteer 1 (thoracic aorta, Fig. 6a) and clinical patients (abdominal aorta, Fig. 6b–d). These are proof of concept examples which demonstrate that, with the proposed 4D Flow-MRI derived CPC-MRA images, it is possible to reconstruct the thoracic and abdominal aortae of a both healthy volunteers and clinical patients. With 4D Flow-MRI, the stent struts of patient 1 were not visible and therefore could not be reconstructed. Further, as this study focuses on the flow lumen, the struts of the Anaconda™ stent were not segmented from the CT images.

Figure 6 compares the standard magnitude images, which come as part of the 4D sequence, against the CPC-MRA images derived from the proposed methodology. For the clinical patients, CT images are also included. Images were acquired from the same locations in the transverse, sagittal, and coronal planes for each modality to permit a direct comparison. As evidenced, the image quality of the magnitude images was very poor due to low SNR and contrast, hence they can generate only a very rough and ambiguous outline of the vessel lumen. Often, the lumen was indistinguishable from surrounding tissue. The CPC-MRA images, however, yield a much clearer lumen with high and uniform signal intensity, which distinctly contrasts against surrounding static tissue and air-filled regions. CT images portrayed a more accurate representation of true and false lumen of the abdominal aorta in patients 2 and 3. This is because a single-VENC MRI sequence was utilized for 4D Flow-MRI and therefore it was not possible to capture flow (and therefore signal intensity) in both the true and false lumen simultaneously. By altering the velocity threshold of the CPC-MRA images, it was possible to retrospectively increase the signal within the false lumen, but the limited spatial resolution of the sequence prohibited delineation between the true and false lumen.

As the temporal CPC-MRA images combine SA, PS, and SD, this ensures all areas of the lumen demonstrate maximum signal intensity, overcoming the temporal lag of blood velocity through cardiac cycle, as distal regions are not underestimated due to low blood velocity. Consequently, segmentation was simple to perform via threshold-based segmentation. It was also possible to increase the signal intensity by decreasing the velocity threshold on cvi42®. A range between 25 and 40 cm s^−1^ produced the best results, with low noise. At < 25 cm s^−1^, it became difficult to distinguish the lumen due to noise, and at > 40 cm s^-1^, there was a risk of underestimating lumen diameter. This range is expected to change according to the initial signal-to-noise ratio of the acquisition sequence, presence and stage of pathology, and the anatomical site of interest.

**Fig. 7:**
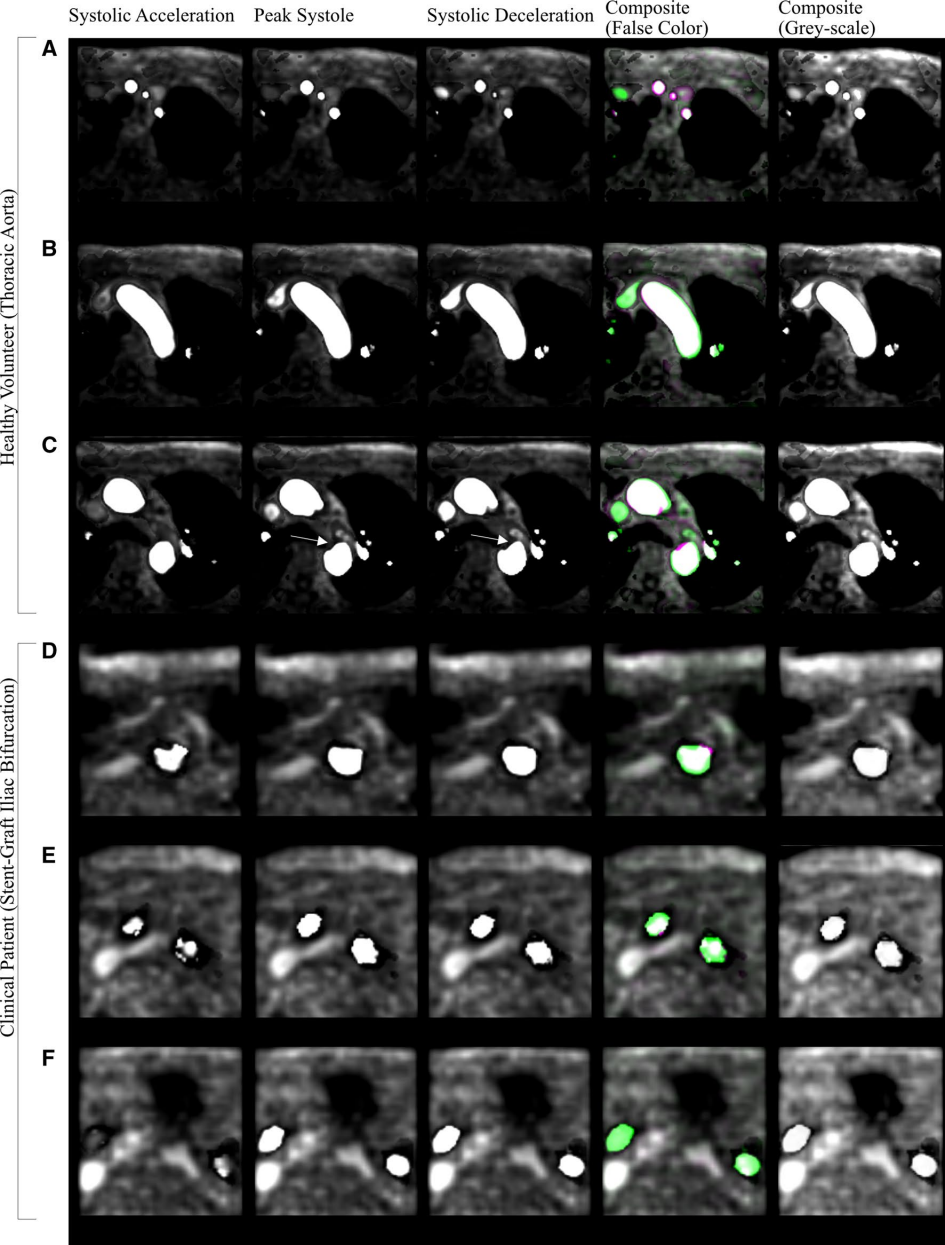
Individual time steps at systolic acceleration, peak systole, and systolic deceleration used to create the CPC-MRA image. False color images are used for visualization while greyscale for segmentation. Healthy volunteer: **A** Supra-aortic branches, **B** Aortic arch, **C** Ascending and descending aorta. Clinical patient 1 with Anaconda™ stent-graft: **D** Abdominal aorta, **E** Common iliac arteries (more proximal), and **F** Common iliac arteries (more distal). White regions in the false color CPC-MRA image show where the three time steps exhibit the same lumen intensity. Magenta and green regions demonstrate where the intensities differ. All images were obtained at a velocity threshold of 25 cm s^−1^ on cvi42®

To highlight the CPC-MRA images in more detail, Fig. 7 was produced with false color on Matlab®. Regions in white indicate areas of the lumen common to each time step, while regions in magenta and green indicate where signal intensity varies during PS and SD respectively. Taking Fig. 7c as an example, this shows the lumen of the ascending and descending aorta. As the phase of the cardiac cycle transitions from SA to SD, a noticeable notch of decreased signal intensity develops in the descending aorta (white arrows). Thus, if images from PS or SD were viewed independently, one may assume this dark region was a kink in the geometry, or simply a narrowed area of the lumen. However, this dark region was not observed during SA. Analysis of the velocity streamlines on cvi42® confirmed this was a region of recirculating flow which began at PS, leading to slow moving flow and reduced velocity magnitude [64]. Hence, the final temporal CPC-MRA image, which combined all three time-steps, filled in this region to create a more representative lumen. Figure 7d–f highlight the CPC-MRA images created from patient 1 at the region of the Anaconda™ stent-graft. Though they are MRI-compatible, the Anconda™ nitinol rings induced noise from metal beam-hardening artifacts during data acquisition which degraded the image quality of the corresponding 4D Flow-MRI CPC-MRA somewhat [65].

### Validation on Patient-Specific Iliac Arteries

The mean tortuosity, radius and curvature were calculated from both the left and right iliac arteries of each of patients 1, 2, and 3, and were grouped (Table 2) according to imaging modality for statistical analysis. Statistical analysis discussed in this work is made in relation to the comparison of CT vs 4D MRI-derived reconstructions and not regarding patient statistics. A Wilcoxon Signed Rank test was performed to evaluate inter-modality differences in radius and curvature along the length of the centerline. No statistically significant inter-modality variation existed for either variable (p > 0.05). There was an insufficient number of data points to determine the statistical significance for differences in vessel tortuosity and bifurcation angle as these were not sampled along the vessel centerlines. The bifurcation angles calculated in VMTK for CT and 4D-Flow MRI were, respectively: 10.1°, 27.6° for patient 1, 78.3°, 65.4° for patient 2, and 62.4°, 57.5° for patient 3. Individual values of tortuosity for CT and 4D-Flow MRI were, respectively: 0.465 ± 0.0657, 0.517 ± 0.0792 for patient 1, 0.325 ± 0.0584, 0.353 ± 0.0359 for patient 2, 0.0792 ± 0.0111, 0.0907 ± 0.00520 for patient 3.

**Table 2:** Mean parameters obtained from the left and right iliac arteries of clinical patients (n = 3) for the CT and 4D-MRI derived models

	Left Iliac			Right Iliac		
	CT	MRI		CT	MRI	
Tortuosity	0.291 ± 0.174	0.307 ± 0.185	–	0.289 ± 0.232	0.333 ± 0.256	–
Curvature (m^−1^)	0.207 ± 0.0704	0.232 ± 0.0757	(p > 0.05)	0.290 ± 0.163	0.279 ± 0.125	(p > 0.05)
Radius (m × 10^−3^)	6.62 ± 0.0691	6.77 ± 0.0256	(p > 0.05)	6.72 ± 0.0725	6.91 ± 0.0426	(p > 0.05)

**Fig. 8:**
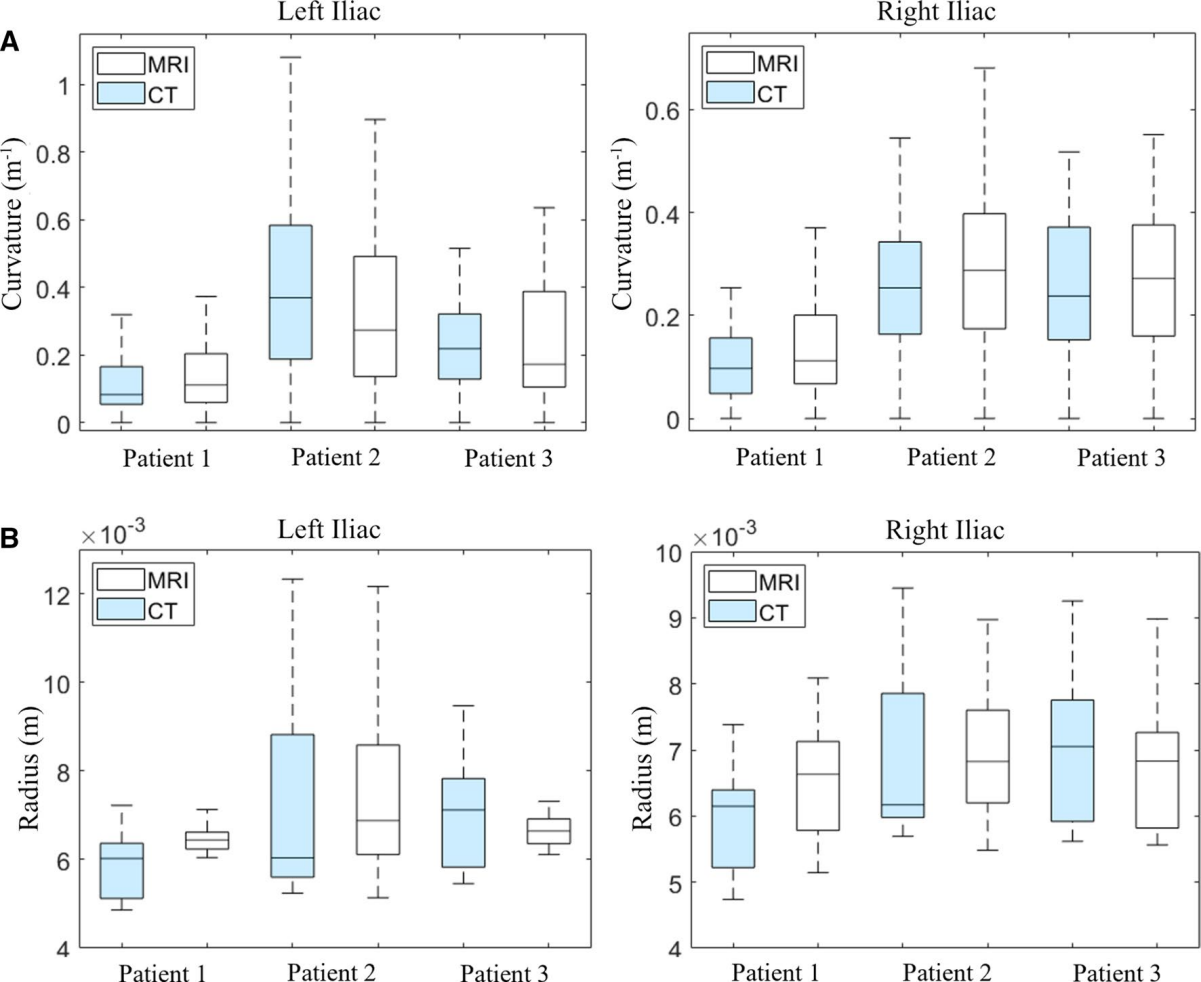
**A** Curvature and **B** radius of the left and right iliac arteries for CT (light blue) and MRI (white) derived models. These parameters were calculated from the vessel centerlines of patients 1, 2, and 3

While there was no statistically significant difference in overall radius and curvature, there was a degree of variability between the CT and 4D-Flow MRI derived models. Figure 8 illustrates the quantitative differences in vessel anatomy. The radial interquartile range (IQR) was consistently smaller for the 4D Flow-MRI models, illustrating less variability in the data compared to CT. Moreover, the median values for 4D Flow-MRI were typically larger than that of CT, indicating that the 4D Flow-MRI derived model may tend to overestimate the vessel radius.

**Fig. 9:**
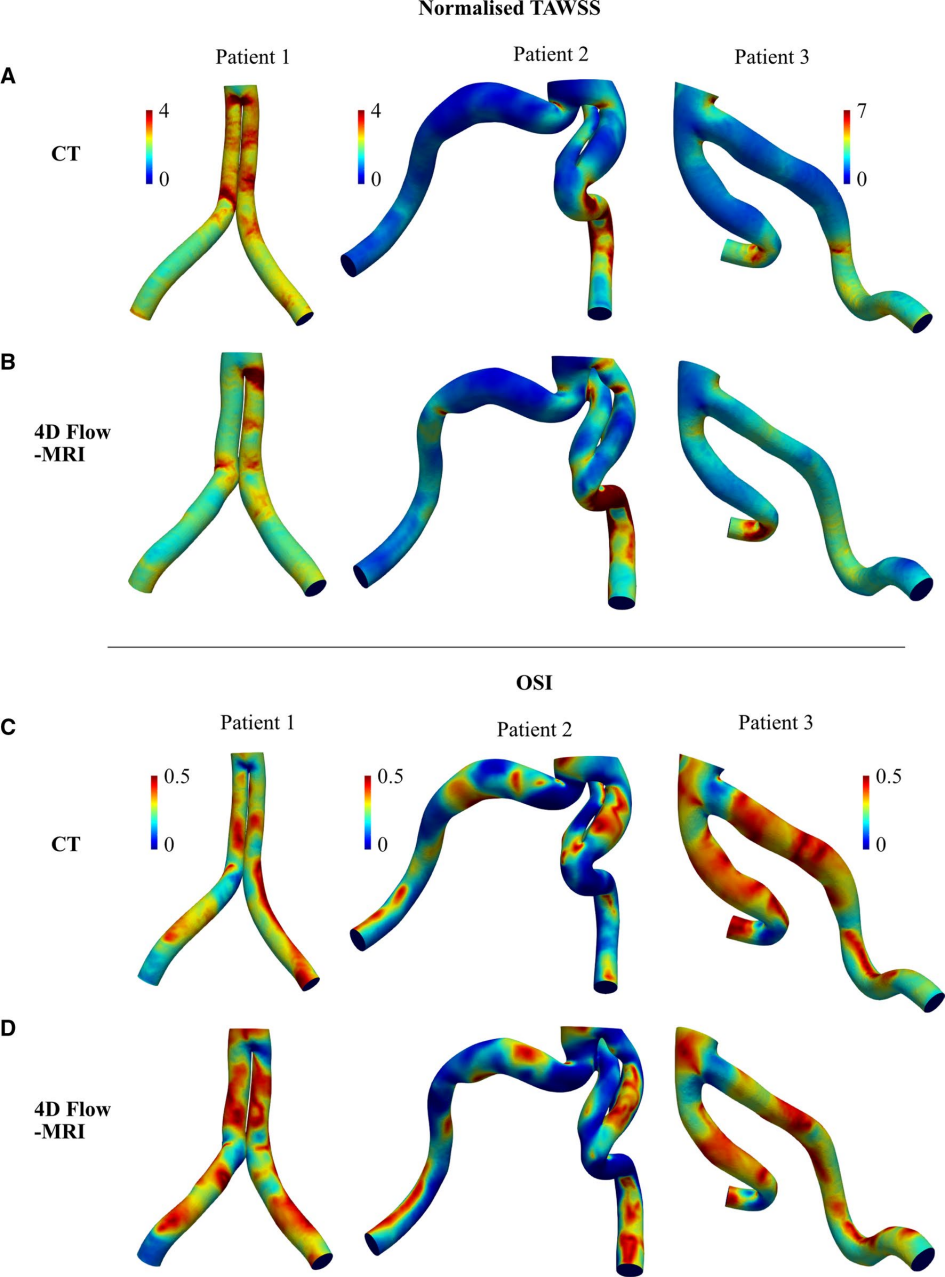
**A**, **B** TAWSS and **C**, **D** OSI distribution across the CT and 4D-MRI derived models of the iliac bifurcation of patients 1 (left), 2 (middle) and 3 (right) calculated from the final cardiac cycle of a patient-specific CFD simulation. TAWSS was normalized with respect to the value calculated at the outlet of the CT cases for each patient (Supplementary Material). Visible for patient 2 is a small region of dissection which extended into the proximal section of the left common iliac artery

Qualitatively, the geometry of the CT and 4D Flow-MRI-derived models were similar, as shown in Figs. 8 and 9. DSC and HD were also included in the analysis as both are widely used to evaluate medical image segmentations to reflect both the lumen size and localization agreement [66, 67]. The DSC was 0.681, 0.736, and 0.736, for patient 1, 2, and 3, respectively. The mean inter-modality HD for patients 1, 2, and 3 was calculated as 5.62 ± 1.44 mm, 7.38 ± 2.56 mm, and 5.18 ± 1.11 mm, respectively. Notably, patient 2 reported the highest pattern of curvature and the largest inter-modality HD. From this preliminary study, it is possible there is a relationship between these metrics, but more data is required to ascertain whether this correlation exists to be considered a clinical limitation.

**Fig. 10:**
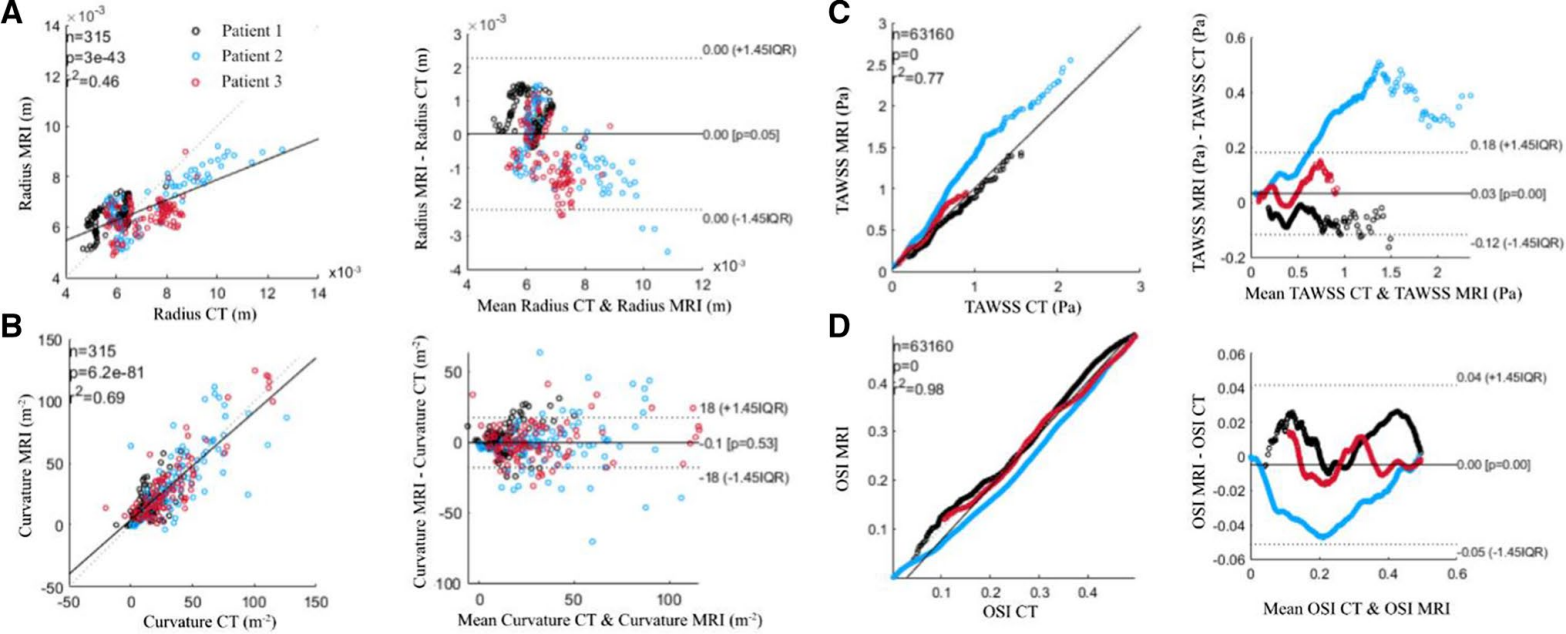
(Left) Correlation and (Right) Bland-Altman plots for patients 1, 2, and 3 at the iliac bifurcation and common iliac arteries, displaying **A** vessel radius, **B** vessel curvature, **C** TAWSS, and **D** OSI

The heterogeneous TAWSS and OSI distribution across the CT and 4D Flow-MRI-derived models are shown in Fig. 9. A Signed Rank test and a Wilcoxon Signed Rank test compared the inter-modality differences in maxima (top 5% of values) and minima (bottom 5% of values) TAWSS across each entire geometry. A significant difference between CT-derived and 4D Flow-MRI derived TAWSS and OSI was present at both extremes within the CFD models (p < 0.05). Localized differences in TAWSS were most apparent in regions of high curvature and at the bifurcation point. The absolute TAWSS values in the CT models ranged from 0.199–1.56Pa, 0.0289–2.16Pa, and 0.0856–0.899 Pa in patients 1, 2, and 3, respectively. Similarly, for the 4D-Flow MRI cases, these values ranged from 0.177–1.43 Pa, 0.0635–2.55 Pa, and 0.0862–0.948 Pa. Figure 10 illustrates a correlation plot and Bland-Altman plot for the vessel radius, curvature, TAWSS, and OSI data obtained from each patient for both CT and 4D Flow-MRI derived models. The Pearson correlation coefficient for each metric was, respectively, 0.46, 0.69, 0.77, and 0.98 (p < 0.05). Due to non-normally distributed data, the Bland-Altman limits of agreement were presented as ± 1.45 $\times$ IQR of the inter-modality difference.

### 4D Flow MRI vs CFD

**Fig. 11:**
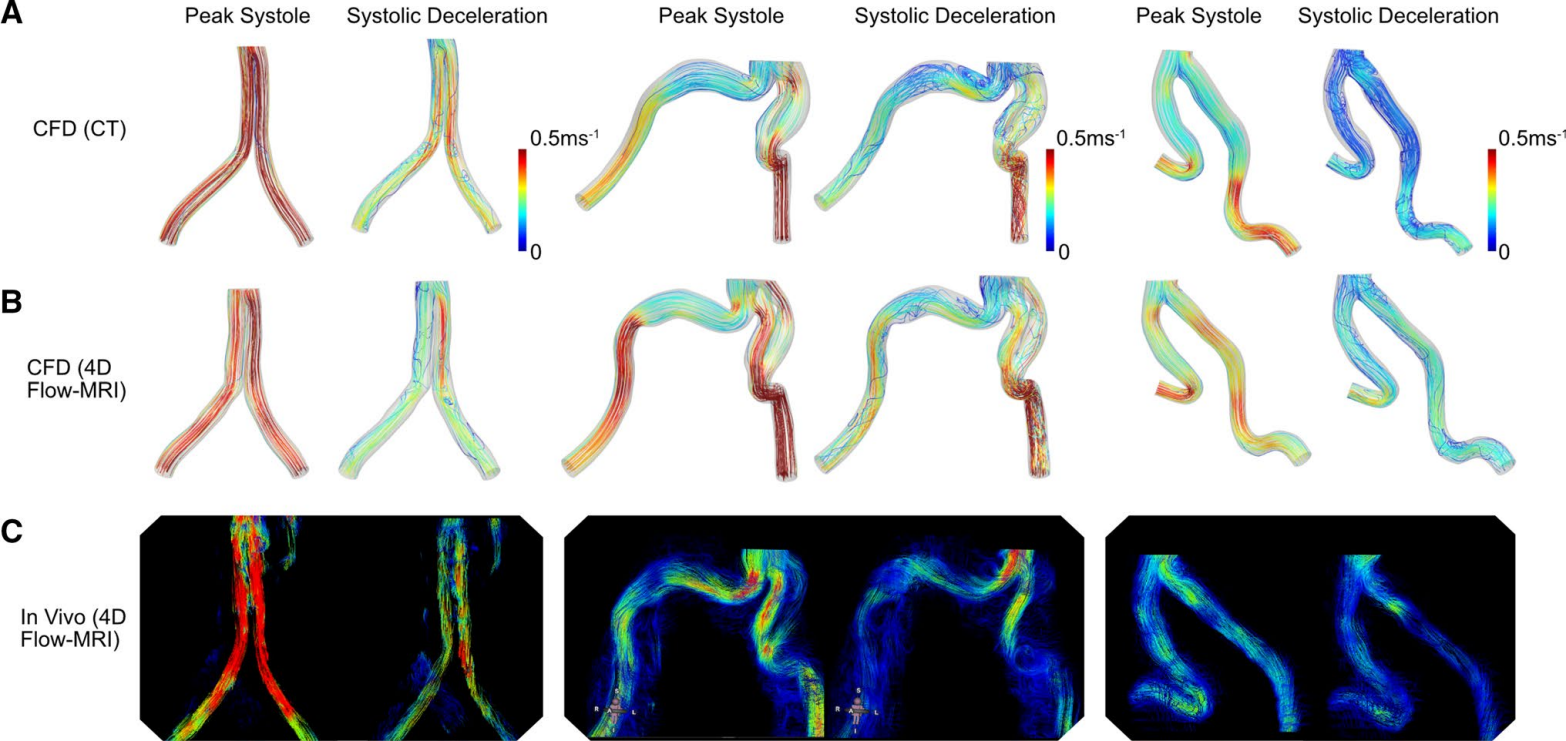
Blood velocity streamlines through the iliac bifurcation and proximal iliac arteries of patients 1, 2, and 3, extracted from **A** CFD models reconstructed from CT images, **B** CFD models reconstructed from 4D Flow-MRI images, and **C** in vivo 4D Flow-MRI (measured in cvi42®). The same value range of 0–0.5 ms^−1^ was used for all cases

The specific 4D Flow-MRI sequence (WIP 785A) used in this study was not calibrated to extract wall shear stress. Therefore, blood velocity streamlines calculated from the CT and 4D Flow-MRI derived CFD models were compared against the in vivo streamlines of velocity magnitude obtained directly from 4D Flow-MRI imaging (Fig. 11). Qualitatively, the overall velocity profiles are similar between the CFD models and in vivo data. However, there were quantitative differences regarding, for example, the maximum through-plane velocities observed at multiple locations throughout the iliac branches. Between patients 2 and 3, the CT-derived CFD models underestimated velocity by 12% and 29% on average during peak systole and systolic deceleration, respectively. Conversely, the MRI-derived models overestimated blood velocity by 9.1% and 0.1%, respectively. Thus, the MRI-derived CFD models demonstrated a smaller discrepancy with the in vivo data. Regarding patient 3, these discrepancies were amplified, where differences in velocity of 48% to 90% were observed between the CFD models and in vivo data, likely due to significant noise and signal artefacts introduced within the 4D Flow-MRI images by the metal-alloy rings of the Anaconda™ stent-graft.

### Sensitivity Analysis

**Fig. 12:**
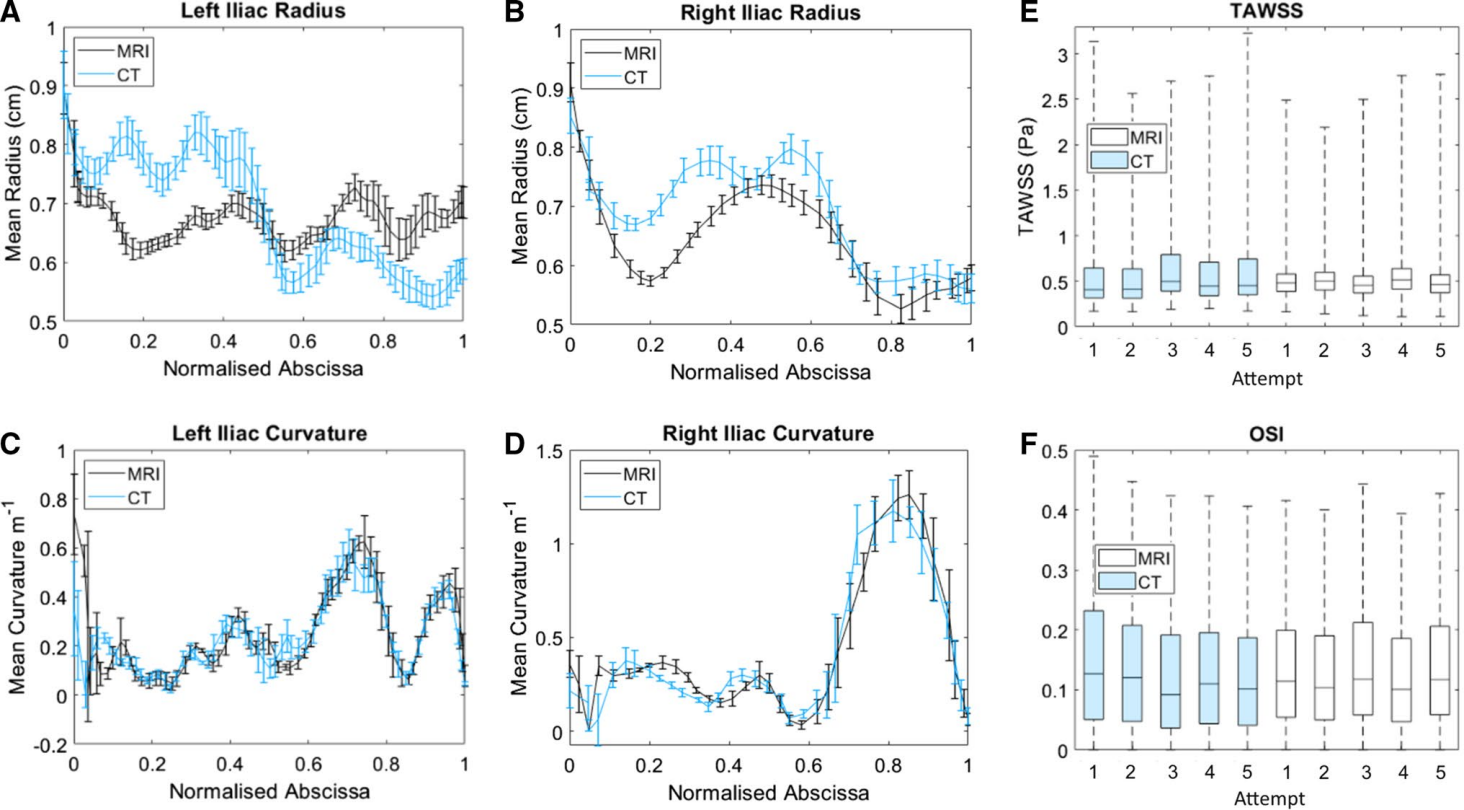
Intra-User Dependence (n = 5) results for vessel geometry and near-wall hemodynamics for both CT (light blue) and 4D Flow-MRI (black) derived models of patient 3. **A** Left and **B** right iliac radius, and **C** left and **D** right iliac vessel curvature, where results are presented as mean ± sd. **E** Box plot of TAWSS and **F** OSI evaluated over the entire iliac geometry for CT (light blue) and 4D Flow-MRI (white)

Intra-user errors, introduced during manual segmentation, were statistically significant for vessel radius and near-wall hemodynamics for both CT and 4D Flow-MRI (p < 0.05). However, there were no significant user-dependent variations concerning the curvature of the vessels (p > 0.05). The localized differences in radius are evident from Fig. 12a and b where, for example, the 4D Flow-MRI derived models underestimated the lumen of the proximal left iliac, and subsequently overestimated the distal portion. Additionally, the variance in radial data in the 4D Flow-MRI models was consistently lower than that of CT.

Intra-user variations at regions of maximum TAWSS was ± 0.29 Pa for CT and ± 0.24 Pa for 4D Flow-MRI (Fig. 12e and f). Consequently, the user may induce an error of up to 0.53 Pa in CFD simulations due to differing perceptions of the lumen during segmentation. Contour plots of TAWSS and OSI distributions for each of the reconstructions can be found in the Supplementary Material. Intra-user CT-derived tortuosity for the left and right iliac was 0.40 ± 0.002 and 0.54 ± 0.014, respectively. Similarly, tortuosity as calculated from the 4D Flow-MRI models was 0.44 ± 0.002 and 0.60 ± 0.013 for the left and right iliac arteries.

## Discussion

The purpose of this study was to develop a novel dataset for the segmentation and reconstruction of patient-specific aortic geometries from retrospective 4D Flow-MRI images. The geometric and CFD-derived hemodynamic parameters obtained from this approach were then compared against CT-derived models, as CT is the gold-standard imaging modality.

### CPC-MRA Composite Images

With the CPC-MRA DICOM stack demonstrated in this study, a clear lumen with uniform signal intensity was observed, distinctly contrasting with surrounding static tissue. Signal intensity within the lumen was proportional to blood velocity magnitude, meaning no ionizing radiation or intravenous contrast agents were required to generate contrast. The boundaries of the vessel lumen were generally well defined, but due to low near-wall velocities which are typical of internal flows, a small region of reduced signal intensity, and hence contrast, was observed around the vessel wall. The creation of these composite image stacks was required to segment the arterial lumen directly from retrospective 4D Flow-MRI data due to the absence of accompanying images such as conventional MRA or PCA.

In some cases, it may be possible to segment the vessel lumen directly from the magnitude images which form part of the 4D Flow-MRI data. In this study however, these images demonstrated poor contrast and low SNR to the extent that in many regions, the lumen was indistinguishable from surrounding tissue. Therefore, it was not possible to segment and reconstruct the vessel geometry directly from these magnitude images. The CPC-MRA images present a significant improvement regarding contrast and signal intensity when compared to the magnitude images (Fig. 5), making the lumen relatively simple to segment. This methodology is therefore beneficial for extracting the lumen for CFD models as an alternative to standard techniques in retrospective datasets. As the final CPC-MRA images were generated by superimposing information on the same slice over multiple time steps, contrast was generated without sacrificing any spatial information along the transverse axis [9, 68, 69].

The temporal CPC-MRA methodology utilizes the same underlying principles as a phase contrast angiogram (PCA), where a phase shift due to the movement of blood is proportional to fluid velocity. These PCA images require prospective planning as vessel contrast and signal intensity is generated during the scan. The 4D Flow-MRI CPC-MRA, however, generated this PCA-type image in a different way. The CPC-PCA was generated from the interpolated 3D velocity profile of retrospective datasets, where these instantaneous velocity profiles were superimposed directly onto the 4D Flow-MRI magnitude images at multiple, user-defined timesteps. This meant the signal to noise ratio of the final angiogram could be controlled post-hoc by the user by simply altering the velocity threshold. Additionally, this postprocessing approach suppresses background noise and reduces the signal from nearby veins because of the slow venous flow. For example, it was possible to increase the velocity threshold to suppress the vena cava and enhance arterial visibility. The opposite is also true, as this approach allows the user to increase signal intensity in branches or regions which experience reduced flow, including aneurysm sacs and the false lumen of an aortic dissection, potentially overcoming a limitation of single-VENC MRI. To validate this however, future work is required to assess this methodology with increased spatial resolution against a multi-VENC sequence [70]. Finally, it is known that in pathological situations involving jet flow, such as aortic dissection, a signal void can appear in the conventional PCA. With the CPC-MRA methodology, regions of jet flow had the opposite effect, as the final signal was enhanced.

The ability to create this temporal CPC-MRA with user-defined time steps is a significant advantage when operating with velocity-based contrast, as regions of recirculating, oscillatory, or regurgitated flow can result in localized drops in signal intensity. As these flow phenomena are often transient, the previous or subsequent time steps, which exhibit a different instantaneous profile, can capture these regions when overlaid as a CPC-MRA. These areas of atypical flow are, however, important clinically, so they can also be analyzed on a time-step by time-step basis within the cvi42® software. Though there are several advantages to this technique, it must be noted that only three user-defined time steps were utilized to create the final CPC-MRA images, out of a total of 20 time points throughout the cardiac cycle. Prior to systolic acceleration, and following systolic deceleration, blood flow, and therefore signal intensity, was too low in magnitude to generate sufficient contrast throughout the lumen. Therefore, reconstruction is constrained only to the mid-systolic phases, meaning information regarding vessel geometry at the end-systolic and diastolic phases was not elucidated. It is possible that this was the result of an overestimated VENC parameter during the MRI imaging sequence, meaning flow was only captured optimally over a limited phase shift range (systolic phases).

### Clinical Relevance

Due to the inherent safety of 4D Flow-MRI, the methodology outlined in this study may be particularly beneficial for the reconstruction of arterial geometry in such patients who have received a stent-graft, as they require serial examinations and cumulative radiation dosages which cannot be avoided with CT, especially in the radiosensitive abdominopelvic region [16, 22]. Additionally, the preliminary functional information may aid in classifying endoleaks and locating any intraluminal tears in cases of aortic dissection, which can be visualized as regions of high velocity jet flow [71–73].

Consequently, 4D Flow-MRI based models and alternative ways to generate luminal contrast may become increasingly sought after. Current data cannot yet demonstrate that this approach yields a more effective assessment when compared to CT imaging. This methodology may also be useful for pregnant patients, where there is a lack of clinical data on the usage of Gadolinium based contrast agents [74–76]. It must be noted however that the metal stent struts were not visible from the 4D Flow-MRI data, meaning this methodology could not yield information on stent integrity, such as fractures, and therefore could not entirely replace CT angiography for post-operative monitoring.

Finally, by utilizing this methodology, it is possible to generate reference models which include both anatomical and functional flow information within the healthy population without ethical concerns. This includes screening of asymptomatic patients, where the 3D anatomical models of otherwise healthy individuals can be created for geometric and CFD-based analysis, as it is accepted that near-wall hemodynamics can be utilized to predict regions of aneurysm formation and future primary entry sites of aortic dissections [77, 78]. When combined with the raw functional information yielded from the same 4D Flow-MRI scan, these models may contribute towards the area of preventative medicine.

### Validation

Validation of the 4D Flow-MRI velocity-derived dataset against the current gold standard, CT angiography, was crucial. A small discrepancy between the geometry of the CT and 4D Flow-MRI-derived models was expected due to the inherent differences in the acquisition of these imaging sequences. The helical CT scan was acquired as a breath-hold scan, in the absence of cardiac (ECG) gating, whilst 4D Flow-MRI was acquired with retrospective ECG and respiratory gating. As such, the CT images represent a snapshot at an arbitrary point within the cardiac cycle, while the 4D Flow-MRI images were created from three well-defined, systolic cardiac phases. It is therefore possible that the 4D Flow-MRI and CT images were captured at slightly different points in the cardiac cycle. This difference may be reflected in the HD and DSC metrics, which were utilized in this study to compare models from two independent imaging modalities scans for each patient. Consequently, a reduced DSC and increased HD was expected, with respect to those same metrics applied to segmentations within a single modality. Nevertheless, literature suggests a good overlap occurs when DSC > 0.7, which was found in the inter-modality comparison of patients 2 and 3, with patient 1 ~ 0.7 [48].

Vessel segmentation was performed manually, so the observer’s interpretation of the lumen generated a degree of geometric variability in the CFD models. Nevertheless, no statistically significant differences in vessel radius or curvature were observed between CT and 4D Flow-MRI-derived models (p < 0.05). Regarding the latter, the lower standard deviation for vessel radius, and lower inter-quartile range for TAWSS, indicate the 4D Flow-MRI-based reconstruction methodology may not elucidate the variability in vessel radius to the same degree as CT. Though no significant difference was found, it must be noted that localized inter-modality differences in the geometric parameters were present, most notably concerning the vessel radius, likely due to the low near-wall velocities mentioned previously.

CFD analysis indicated these small differences in vessel geometry amplified any differences in the blood flow regime. This resulted in statistically significant inter-modality differences in near-wall hemodynamics at the upper and lower extremes of TAWSS and OSI (p < 0.05). For example, a further analysis reveals the regions of increased inter-modality TAWSS differences are spatially correlated to regions of increased radial disparities which subsequently alter blood velocity, and therefore TAWSS, for a given flow rate. These discrepancies in vessel radius have a marked impact on the resultant hemodynamics because blood velocity is non-linearly related to radius. However, Fig. 10 demonstrates that there still exists a strong correlation between the CT and 4D Flow-MRI derived TAWSS and OSI, where R^2^ is 0.77 and 0.98 respectively. Additionally, Fig. 11 indicates that the proposed methodology to create images based on retrospective 4D Flow-MRI data does not systematically underestimate or overestimate the lumen when compared to CT. This can be inferred since blood velocity is not consistently higher or lower in the 4D Flow-MRI-derived CFD models when compared to the CT-derived CFD models. It is important to note, however, that a larger study is required validate this claim.

As the upper and lower extremes of TAWSS and OSI are important in clinical applications, the inter-modality discrepancies must be highlighted [58, 63]. Within this study, these regions differed by only 0.39 Pa and 0.035 Pa, respectively, between CT and 4D Flow-MRI. Further, the sensitivity analysis determined that the user may be responsible for up to ~ 0.53 Pa of this discrepancy, due to variations in lumen interpretation. As such, the true inter-modality difference in TAWSS may be negligible. These differences are also low in comparison to the high shear stresses (> 5–10 Pa) which can induce platelet activation, and therefore may not be clinically significant. However, these discrepancies should be noted when assessing the risk of thrombosis [63].

#### CFD vs In Vivo 4D Flow-MRI

Regarding blood velocity, the MRI-derived CFD models demonstrated a superior degree of qualitative similarity to the in vivo data when compared to the CT-derived models. However, it is important to acknowledge that disparities persisted, which can be attributed to various factors. Primarily, manual errors introduced by the operator during vessel segmentation likely contributed largely to this, as these were regions of challenging anatomy, encompassing features such as aortic dissection and a stent-graft. In the case of the latter, the presence of metal components in the stent-graft likely resulted in local field disruptions, thus causing significant artifacts during the acquisition of 4D Flow-MRI [79]. Aside from geometric differences and signal artifacts, it is important to consider the inherent differences between 4D Flow-MRI imaging and CFD modeling. In recognition of these differences, quantitative discrepancies between the two approaches are commonly observed in literature [80–83]. Firstly, the spatiotemporal resolution is very high for the CFD models, but very low for the in vivo scan sequence. This mismatch of resolution is known to introduce differences in resultant blood velocity, especially in regions of increased flow [80, 81]. Secondly, the scan sequence utilized in this study exhibited a coarse resolution of 3.6 × 2.4 × 2.6 mm^3^, though literature suggest a minimum resolution of 1.5 × 1.5 × 1.5 mm^3^ is desirable [82]. Accordingly, it is reasonable to assume that the lower-resolution 4D Flow-MRI scans can induce errors in the flow field since the results relies increasingly on data interpolation. Due to the retrospective nature of the dataset, this could not be refined [82]. Thirdly, it should be recognized that the 4D Flow-MRI scan did not incorporate isotropic spatial resolution, thereby causing the resulting velocity measurements to be directionally dependent, unlike the CFD models [82]. Finally, the assumption of rigid walls and the absence of the proximal aorta in the numerical domain likely contributed to the differences in CFD vs *in vivo* velocity profiles. To validate these statements however, a larger study is required.

### Limitations and Future Work

There were several limitations to this study, mainly due to the retrospective nature of the 4D Flow-MRI data set. Firstly, a low number of geometries (n = 3) were used for validation purposes due to limited patient data. More data are required to ascertain the reliability of the novel methodology proposed for dataset generation. This study, however, was intended as a proof-of-concept analysis to demonstrate how the novel methodology may contribute towards the reconstruction of 4D Flow-MRI images for use in CFD, particularly for retrospective datasets in the absence of standard images such as MRA and PCA.

However, with numerous data points for each modality for both radius and curvature, it was possible to determine statistical significance in relation to the comparison of CT vs 4D Flow-MRI-derived reconstructions, and not regarding patient statistics. Additionally, validation was restricted to healthy and stented regions of the clinical patients as the 4D Flow-MRI sequence utilized in this study was not optimized for visualization of the false lumen. To include larger, more complicated regions of pathology, future studies would require multi-VENC 4D Flow-MRI imaging, which can capture significantly different velocities within the same scan. Further, the limited spatial resolution of the research-based 4D Flow-MRI sequence used to acquire the images in this study may have affected the validation study. Due to the intrinsic resolution of the dataset, this could not be improved.

An unsteady parabolic profile was prescribed at the inlet of the CFD domains. It was not possible to extract the decomposed spatial velocity profile from cvi42®, and therefore this could not be prescribed directly as a Dirichlet boundary condition at the inlet.

The rigid wall assumption affects the accuracy of clinically relevant CFD-derived hemodynamic metrics including TAWSS and OSI [83, 84]. By omitting the compliance of the native arteries for example, TAWSS is generally overestimated, particularly due to elevated wall shear stress at peak systole [83].

Future work will integrate 4D Flow-MRI time-resolved data regarding vessel wall motion into the numerical model to improve the accuracy of the CFD simulations by creating a moving boundary method (MBM) model [83, 84]. To do so, the geometry of the vessel must be captured at all stages of the cardiac cycle. Therefore, future prospective studies will iteratively reduce and optimize the VENC parameter to capture blood velocity, and therefore signal intensity during the late-systolic and diastolic phases. These MBM simulations can then be performed at a substantially reduced computational cost in comparison to fluid-structure interaction (FSI) models, account for external loads applied by surrounding tissue, and utilize data which is measurable in vivo, thus limiting the required assumptions [83, 84].

## Conclusion

In this study, a novel dataset was created from multiple 4D Flow-MRI-derived images at reproducible time steps throughout the cardiac cycle, yielding a temporal CPC-MRA image dataset. This study presents proof-of-concept examples of how functional 4D Flow-MRI data can be retrospectively translated to generate 3D anatomical models for geometric analysis and CFD in healthy and stent-graft cases. The blood velocity-based approach yielded uniform signal intensity throughout the lumen, clearly contrasting with surrounding static tissue while preserving the 3D relationships of overlapping vascular anatomy. Fundamentally, the outlined methodology required no ionizing radiation or intravenous contrast and could be performed on retrospective data sets. This processing of the 4D Flow-MRI data prepares it for most image segmentation methodologies, from thresholding to machine learning and convolutional neural networks. Finally, the proposed pipeline for 4D Flow-MRI derived image creation may be used for 3D model generation of healthy and stented aortae in cases where 4D Flow-MRI is available, for example when screening for aortic disease, pregnant women, or children.

### Supplementary Information

Below is the link to the electronic supplementary material.

Supplementary file1 (DOCX 2465 kb)

## Declarations

### Animal Studies

No animal studies were carried out by the authors for this article
