# Supplementary material for: Reconstruction and Validation of Arterial Geometries for Computational Fluid Dynamics Using Multiple Temporal Frames of 4D Flow-MRI Magnitude Images
Source: Cardiovasc Eng Technol. 2023 Aug 31;14(5):655–76. doi: 10.1007/s13239-023-00679-x (PMC10602980; doi:10.1007/s13239-023-00679-x)
Supplement: Supplementary file 1 — Supplementary file1 (DOCX 2465 kb) [file 13239_2023_679_MOESM1_ESM.docx]

Supplementary Material: Reconstruction and Validation of Arterial Geometries for CFD using Multiple Temporal Frames of 4D Flow-MRI Magnitude Images

*Cardiovascular Engineering and Technology*

Scott MacDonald Black^1^, Craig Maclean^2^, Pauline Hall Barrientos^3^, Konstantinos Ritos^4,5^, Asimina Kazakidi*^1^

^1^Department of Biomedical Engineering, University of Strathclyde, Glasgow, UK. ORCID (Asimina): 0000-0001-7124-4123

^2^Research and Development, Terumo Aortic, Glasgow, UK. ORCID: 0000-0002-7941-813X

^3^Clinical Physics, Queen Elizabeth University Hospital, NHS Greater Glasgow & Clyde, Glasgow, UK. ORCID: 0000-0002-1563-233X

^4^Department of Mechanical and Aerospace Engineering, University of Strathclyde, Glasgow, UK. ORCID: 0000-0001-6334-6680

^5^Department of Mechanical Engineering, University of Thessaly, Volos, Greece. ORCID: 0000-0001-6334-6680

*** Correspondence:** Asimina Kazakidi. Asimina.kazakidi@strath.ac.uk

# Mesh Generation

## Boundary Layer

The initial boundary layer height, y, on all computational meshes was calculated using Eq.1.

$y=\frac{y^{+}v}{u_{\tau}}$ *(1)*

Where $y^{+}$=1, $\left( v=\frac{\mu}{\rho} \right)$ was the kinematic viscosity, and $u_{\tau}$was the friction velocity (Eq.2).

$u_{\tau}=\sqrt{\frac{\tau_{\omega}}{\rho}}$ *(2)*

Where $\rho$ was the density of blood and $\tau_{\omega}$ was wall shear stress. To estimate $\tau_{\omega}$, Eq.3 was calculated from the skin friction coefficient, $C_{f}$, and free stream velocity, $U_{\infty}$.

$\tau_{\omega}=\frac{1}{2}C_{f}\rho U_{\infty}^{2}$ *(3)*

For this study, $U_{\infty}$ was defined as the maximum velocity (U_Systole_) of a single cardiac cycle. [1] Further, the Schlichting equation (Eq.4) was employed to calculate the skin friction coefficient as a function of Reynolds number (Re) at peak systole. [2]

$C_{f}={[2 {log}_{10}\left( Re \right)-0.65]}^{-2.3}$ *(4)*

## Grid Convergence Study for Mesh Independence

To investigate the spatial convergence of the computational fluid dynamics (CFD) simulations and achieve mesh independence for wall shear stress (WSS), a grid convergence study was performed on the geometry of patient 3. A steady state Reynolds averaged Navier Stokes (RANS) simulation was therefore performed with a standard k-$\omega$ turbulence model and constant inlet velocity (U_Systole_) until continuity residual values met the convergence criteria of 1e-6. Turbulent intensity (Eq.5) and hydraulic diameter, D_H_, were prescribed at the inlet.

$I=0.16\left( Re_{DH} \right)^{- \frac{1}{8}}$ *(5)*

As the number of mesh elements are increased, the spatial discretization error asymptotically approaches zero. First, the order of convergence was calculated as per Eq.6:

$p=\ln\left( \frac{f_{3}-f_{2}}{f_{2}-f_{1}} \right)\frac{1}{ln(r)}$ *(6)*

Where $f_{1}$, $f_{2}$, and $f_{3}$ are the surface integral values of wall shear stress at the bifurcation for the coarse, medium, and fine mesh densities.

A Richardson extrapolation (Eq.7) was then performed to estimate the true value of the WSS parameter, based on the order of convergence previously calculated.

$f_{h=0}=f_{3}+\frac{f_{1}-f_{2}}{r^{P}-1}$ *(7)*

Then, the grid convergence index (GCI) was then calculated for each refinement level, as per Eq.8.

$GCI=\frac{F_{s}\left| e \right|}{r^{p}-1}$ *(8)*

Where $F_{s}$ =2 is the safety factor, and $\left| e \right|$ is the error between the refinement levels. To then ensure grid convergence is being evaluated within the asymptotic range, thus approaching a converged answer, the following relationship must hold true [3].

$$\frac{{GCI}_{2,3}}{{r^{P}\times GCI}_{1,2}}\cong1$$

The initial coarse mesh of 250,000 elements was generated and subsequently refined 4 times up to a density of 4M elements (Table S1). The ratio of refinement for each step was maintained as a constant value of 2, therefore doubling the number of elements each time. At each level of mesh refinement, the solution lies within the asymptotic region of convergence. Further, at the finest mesh density of 4M elements, a Richardson extrapolation value $f_{h=0}=$2.74x10^-4^ was obtained.

**Table S1**: Grid convergence study to determine mesh independence for patient 3

| Mesh Density (M) | WSS Integral | % Difference from Coarse | % Difference from Richardson Extrapolation (2.74x10^-4^) |
| --- | --- | --- | --- |
| 0.25 | 0.000252 | - | 8.37 |
| 0.5 | 0.000258 | 2.08 | 6.02 |
| 1 | 0.000261 | 3.29 | 4.86 |
| 2 | 0.000262 | 3.82 | 4.48 |
| 4 | 0.000263 | 4.27 | 4.10 |

A 1M element mesh was therefore utilized for all subsequent CFD simulations within the study as the error calculated with respect to the Richardson extrapolation was <5%. Successive doubling of the mesh density after this point only improves the error by ~0.5% each time, meaning these small improvements after 1M elements were not worth the significant increases in computational cost.

# TAWSS Normalization

The TAWSS distribution for each patient was normalized with respect to TAWSS at the left iliac outlet, calculated analytically as follows.

$\tau_{\omega}=-\mu\frac{du}{dy}$ *(13)*

Where

$\frac{u}{u_{max}}=1-\left( \frac{2y}{h} \right)^{2}$ *(14)*

Where h is the radius, $y=h/2$, the velocity at the apex of the fully developed flow, $U_{max}=\frac{3}{2}U_{inlet}$, and $\mu$=0.004 Pa s.

$\frac{du}{dy}=\frac{-8u_{max}y}{h^{2}}$ *(15)*

$\tau_{\omega}=\frac{8\mu u_{max}y}{h^{2}}$ *(16)*

At each point within a single cardiac cycle, the instantaneous $\tau_{\omega}$ was calculated at the inlet of the CT CFD model on Matlab® to determine a time-averaged value at the inlet, $\tau_{\bar{\omega}}=\int_{0}^{T} \tau_{\omega}dt$*.* For patient 1, 2, and 3, $\tau_{\bar{\omega}}$ was equal to 0.439Pa, 0.600Pa, and 0.248Pa respectively.

# Instantaneous Reconstructions

Figure 13 illustrates the reconstructed vessel of the healthy volunteer at multiple, independent time points throughout the cardiac cycle. The reconstructed geometry is different at each time point as the velocity magnitude, and therefore signal intensity within the lumen, changes throughout the cardiac cycle. Prior to SA, only the proximal ascending aorta could be reconstructed. Following SD, the signal intensity gradually decreased and disappeared, and the reconstruction was not representative of the aorta. The combination of SA, PS, and SD generated the optimal reconstruction.


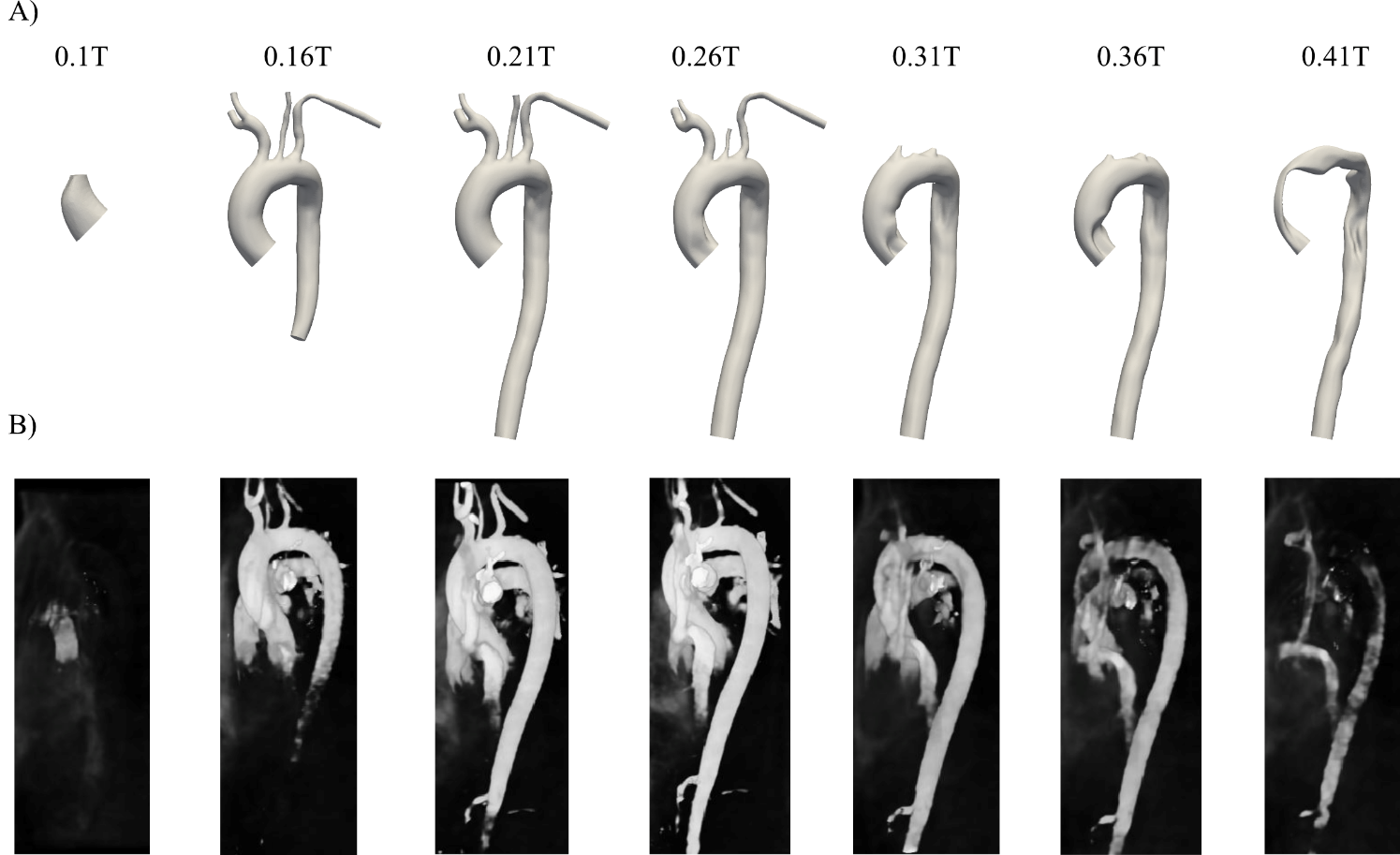


**Fig. 13** A) Reconstructed geometry of the thoracic aorta of the healthy volunteer, and B) rendered image created from the velocity-based 4D Flow-MRI signal at multiple time steps during the cardiac cycle. Lumen contrast was generated from the instantaneous velocity magnitude profile (retrospectively overlaid as in Section 2.1.4) at each time step**.** T is the period of the cardiac cycle (0.21T: Systolic acceleration; 0.26T: Peak systole; 0.36T: Systolic deceleration.

# Sensitivity Analysis: TAWSS and OSI

TAWSS distributions (Figure 14) were obtained from computational fluid dynamics (CFD) analysis of patient 3 during the sensitivity analysis to determine the effect of intra-user segmentation errors. Segmentation and reconstruction of the geometry was performed 5 times for both computed tomography (CT) and 4D Flow magnetic resonance imaging (4D Flow-MRI). Qualitatively, the TAWSS distribution remains relatively constant within each modality, though larger inter-modality discrepancies are apparent. Elevated regions of TAWSS are visible at the point of bifurcation and regions of increased curvature.

A)

2

**TAWSS**


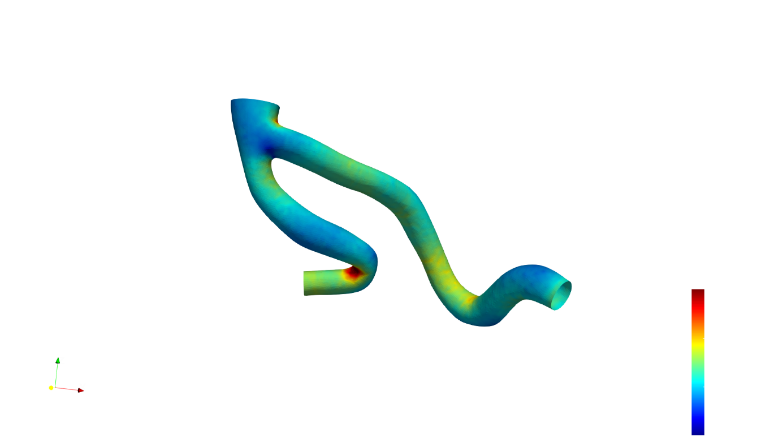

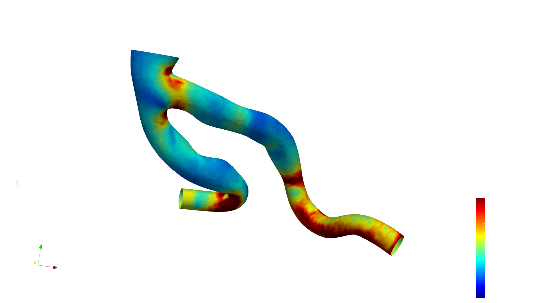

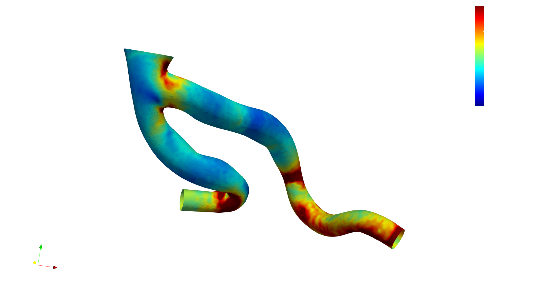

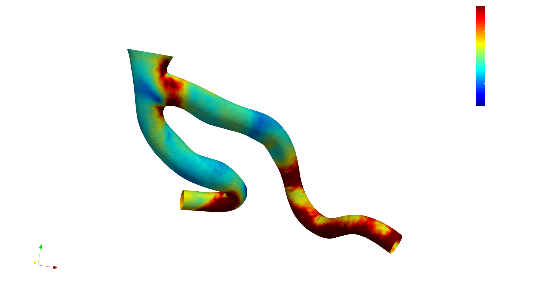

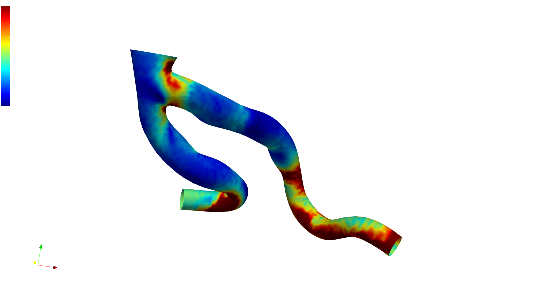

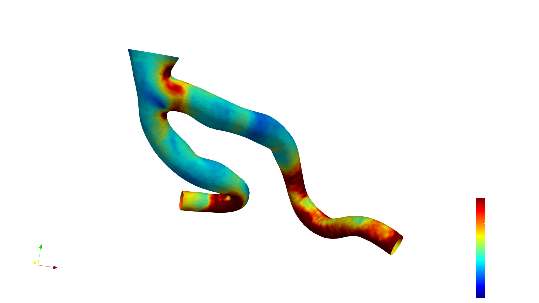


0

B)

*
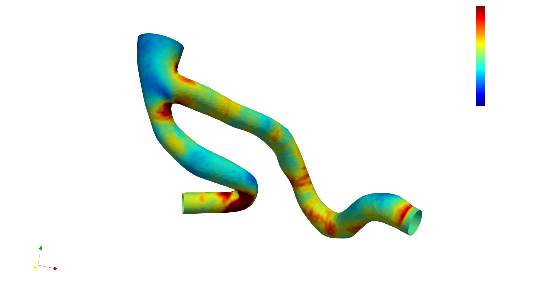

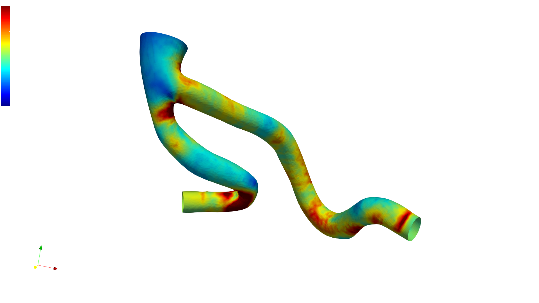

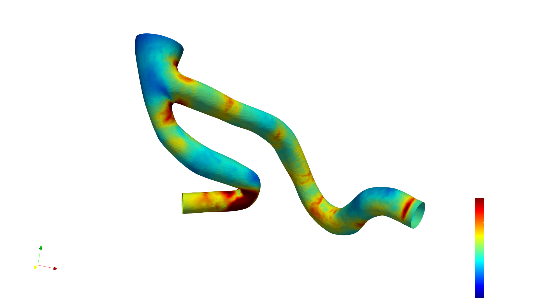

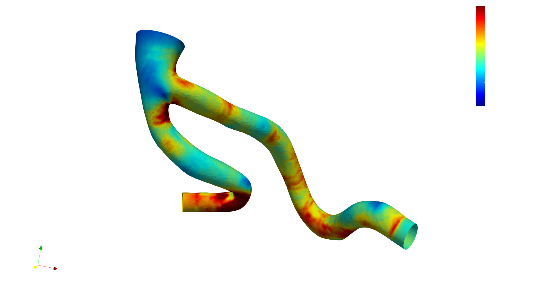

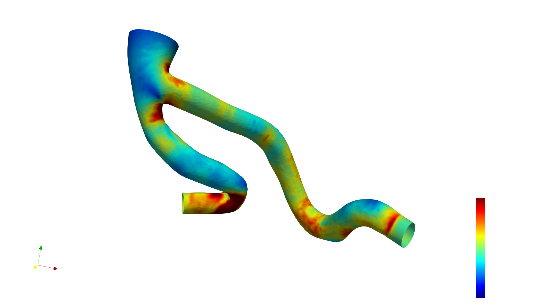
*

**Fig 14** TAWSS distribution for patient 3, obtained via CFD simulations of the A) CT and B) 4D Flow-MRI derived geometries. Each modality was segmented and reconstructed 5 times

Similarly, OSI distributions obtained from the sensitivity analysis are visible in Figure 15. Again, minimal qualitative differences exist within each modality. However, differences between the CT and 4D Flow-MRI cases are more apparent. Elevated regions of OSI occur at areas of low TAWSS.

**OSI**

0.5

A)


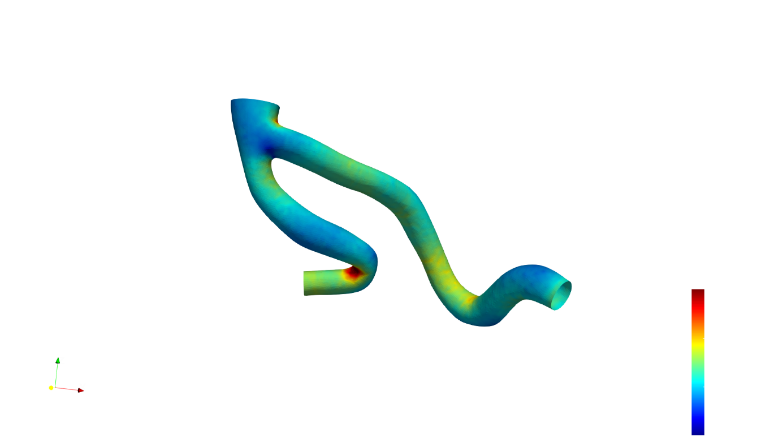

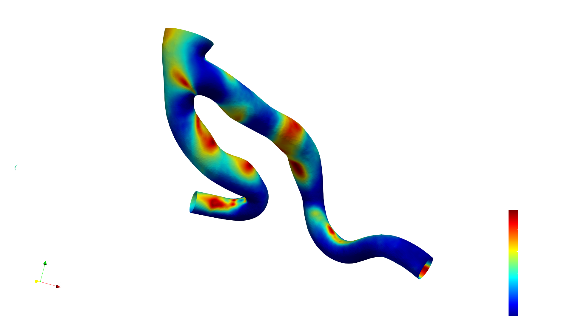

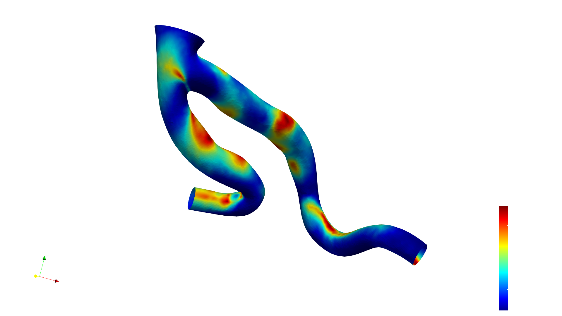

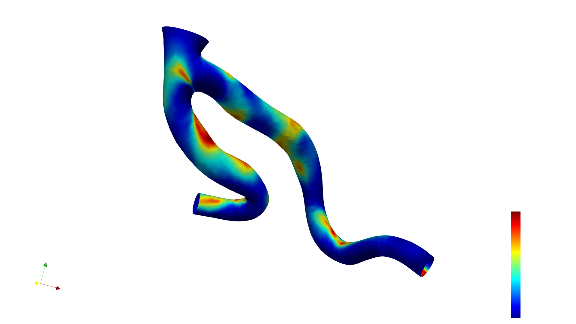

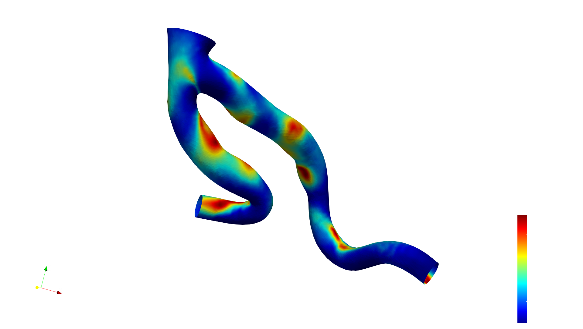

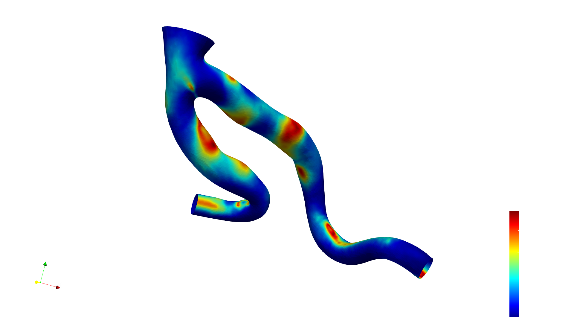


0

B)


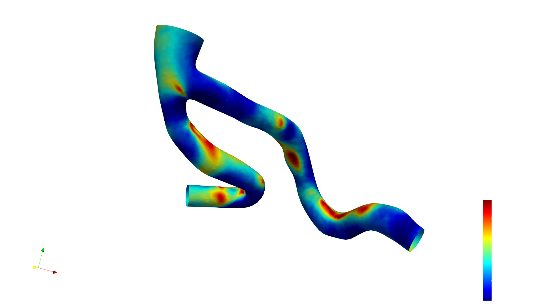

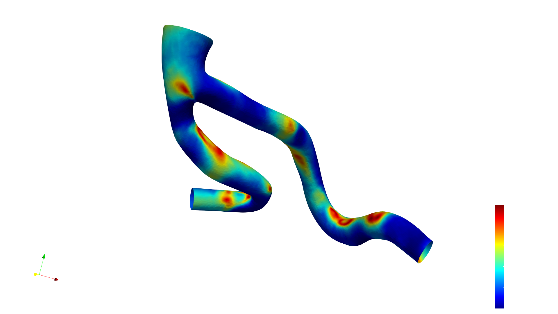

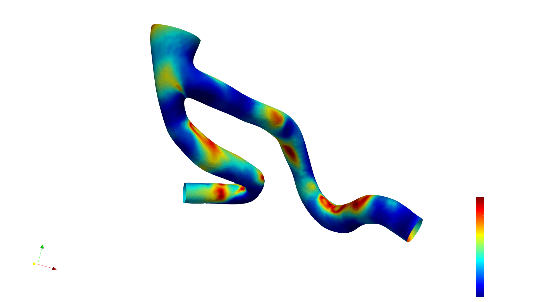

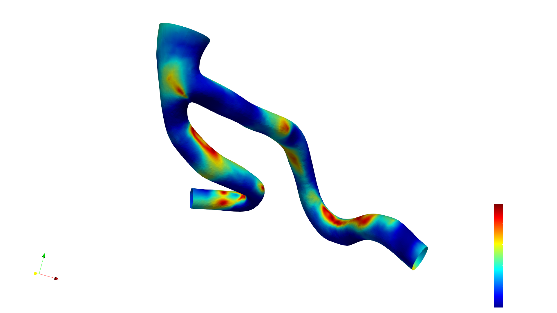

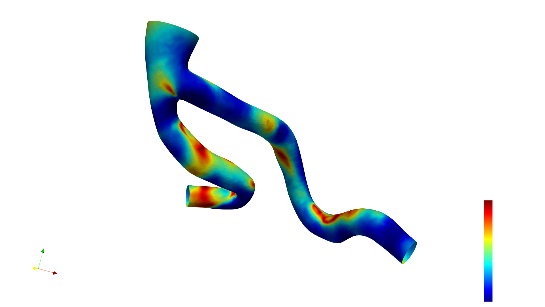


**Fig 15** OSI distribution for patient 3, obtained via CFD simulations of the A) CT and B) 4D Flow-MRI derived geometries. Each modality was segmented and reconstructed 5 times

# Raw Centerline Data

Figure 16 illustrates the raw centerline data which was extracted from the 4D Flow-MRI and CT-derived models at the iliac bifurcation and the proximal region of the common iliac arteries of patient 1, 2, and 3. This radius and curvature data was calculated on the Vascular Modelling Toolkit® (VMTK®). Between 37-70 datapoints form the curves for each common iliac artery, depending on the length of the vessel centerline. Patient 2 exhibited a region of dissection in the left common iliac artery, resulting in a true lumen (TL) and false lumen (FL), so radius, R, is presented as R=R_TL_+R_FL_.


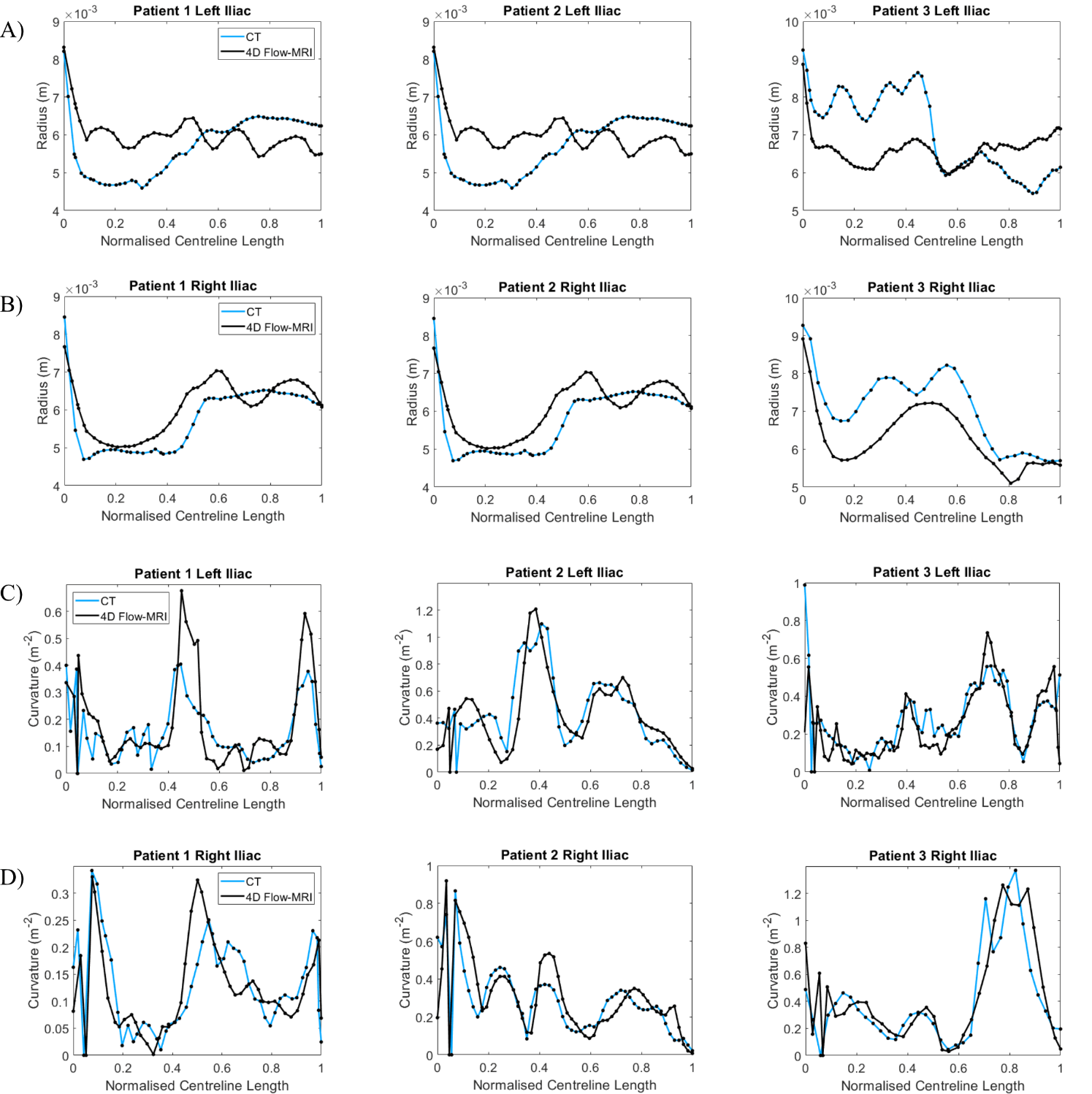


**Fig 16** Vessel radius and curvature at discrete points along the length of the vessel centerlines of the common left and right iliac arteries for patient 1, 2, and 3. These values were extracted from the CT-derived (blue) and 4D Flow-MRI-derived (black) models.

# Statistical Analysis

Statistical analysis was performed on Minitab® to investigate the geometric and hemodynamic data during validation of the 4D Flow-MRI-derived models against CT-derived models, and for the intra-user sensitivity analysis. To determine normality of the data distribution, an Anderson Darling normality test was employed, which concluded both the geometric and hemodynamic data distributions were non-normal (p<0.05). Consequently, non-parametric statistical tests were employed as these perform well with skewed distributions and those which are better represented by the median instead of the mean. Further, as the 4D Flow-MRI and CT images were acquired from the same patients, the data was considered dependent. Therefore, a combination of Signed Rank tests and 1-Sample Wilcoxon tests were utilized. hereafter, the difference between the dependent samples was calculated and the distribution of these differences were analyzed to ensure symmetry. If symmetry was not observed, a Johnson Transform was applied to transform the data and generate a more symmetrical distribution. If a transform was not possible, a Signed Rank Test was employed instead of a 1-Sample Wilcoxon. Outliers were removed on all data sets which were detected using the Grubbs Test. One exception to this method was the sensitivity analysis data for vessel curvature, which could be transformed to generate a normal distribution prior to hypothesis testing. In this case, a paired t-test was utilized. In all cases, the significance level, α=0.05.

# References

| [1] | J. Xing, in *Fluid-Solid Interaction Dynamics: Theory, Variational Principles, Numerical Methods, and Applications*, Southampton, United Kingdom, Academic Press, 2019, pp. 487-575. |
| --- | --- |
| [2] | H. Schlichting, Boundary-layer Theory, New York : London: McGraw-Hill, 1979. |
| [3] | N. Baker, G. Kelly and P. D. O'Sullivan, "A grid convergence index study of mesh style effect on the accuracy of the numerical results for an indoor airflow profile," *International Journal of Ventilation,* vol. 19, no. 4, pp. 300-314, 2019. |
